# The safety and efficacy of non-typeable Haemophilus influenzae and Moraxella catarrhalis vaccine in chronic obstructive pulmonary disease: a systematic review and meta-analysis of randomized controlled trials

**DOI:** 10.3389/fmed.2025.1572726

**Published:** 2025-04-04

**Authors:** Tiankui Shuai, Jing Liu, Meijun Dong, Peng Wu, Lu Zhang, Zhouzhou Feng, Wenqiang Li, Jian Liu

**Affiliations:** ^1^Department of Emergency Critical Care Medicine, The First Hospital of Lanzhou University, Lanzhou, Gansu, China; ^2^The First Clinical Medical College, Lanzhou University, Lanzhou, Gansu, China; ^3^Outpatient Department, The Second Affiliated Hospital of Xi'an Medical University, Xi'an, Shanxi, China; ^4^Department of Internal Medicine, Wenxian First People’s Hospital, Longnan, Lanzhou, Gansu, China; ^5^KeyMed Biosciences Inc., Chengdu, Sichuan, China; ^6^Gansu Provincial Central Hospital, Gansu Provincial Maternity and Child-Care Hospital, Lanzhou, Gansu, China

**Keywords:** chronic obstructive pulmonary disease, non-typeable Haemophilus influenzae, Moraxella catarrhalis bacteremia, vaccine, efficacy and safety, systematic review, meta-analysis

## Abstract

**Background:**

Non-typeable Haemophilus influenzae (NTHi) and Moraxella catarrhalis (Mcat) are major pathogens implicated in bacterial exacerbations of chronic obstructive pulmonary disease (COPD). Their involvement contributes to antibiotic resistance and poses significant immune challenges, underscoring the need for targeted vaccine strategies. This systematic review and meta-analysis assessed the safety and efficacy of NTHi-Mcat/NTHi vaccines in COPD patients.

**Research design and methods:**

Randomized controlled trials (RCTs) assessing the safety and efficacy of NTHi-Mcat/NTHi vaccines for COPD were systematically searched across four databases (PubMed, CENTRAL, Embase, and Medline) from inception to October 2024. Meta-analyses were conducted using random-effects or fixed-effects models, with subgroup analyses to investigate possible sources of heterogeneity.

**Results:**

This analysis included eight RCTs involving 1,574 participants, primarily conducted in Europe (*n* = 3) and Australia (*n* = 2), with interventions administered orally or intramuscularly at varying frequencies (twice or three times). The Meta-analyses revealed that the NTHi-Mcat/NTHi vaccine did not affect the incidence of acute exacerbations of COPD (relative risk (RR): 1.02, 95% confidence interval (CI): 0.76 to 1.36), all-cause mortality (RR: 0.91, 95% CI: 0.38 to 2.21), and hospitalization rate (RR: 0.50, 95% CI: 0.09 to 2.77). Regarding safety, the NTHi-Mcat/NTHi vaccine did not significantly increase the risk of serious adverse events (RR: 1.00, 95% CI: 0.84 to 1.19) or grade 3 serious events (RR: 1.20, 95% CI: 0.93 to 1.53). However, it was associated with a higher risk of local and systemic reactions, including pain (RR: 5.33, 95% CI: 1.98 to 14.33), swelling (RR: 12.15, 95% CI: 4.67 to 31.67), redness (first dose: RR: 12.74, 95% CI: 3.48 to 46.59; second dose: RR: 11.55, 95% CI: 3.90 to 34.22), headaches (RR: 1.20, 95% CI: 1.00 to 1.43), erythema (RR: 15.38, 95% CI: 5.64 to 41.92), and fever (after the second dose: RR: 2.33, 95% CI: 1.24 to 4.38).

**Conclusion:**

Although the NTHi-Mcat/NTHi vaccines were well-tolerated in COPD patients, they did not significantly reduce the risk of exacerbations or mortality. These findings suggest that further research is needed to validate these results and identify potential subgroups that may derive clinical benefit.

**Systematic review registration:**

The study was registered in PROSPERO (ID: CRD42023381488).

## Introduction

Chronic obstructive pulmonary disease (COPD) is a leading cause of morbidity and mortality worldwide, and has a significant and growing economic and social burden (1). Among the 55.4 million deaths worldwide in 2019, COPD is the second and third leading cause of death, accounting for 11 and 6% of total deaths, respectively (2, 3). In the United States, the cost of COPD is projected to grow over the next 20 years, with a projected annual cost of $800.9 billion or $40 billion (4, 5). The Global Burden of Disease (GBD) was significantly higher compared to that in 2010 and 2014, especially for moderate to severe cases (5, 6). The prevalence and burden of COPD are expected to increase in the coming decades because of the continued exposure to COPD risk factors and an aging population (7, 8). Furthermore, individuals with COPD are at a higher risk of experiencing acute exacerbation of chronic obstructive pulmonary disease (AECOPD) (9). The pathology of AECOPD is mainly associated with increased airway inflammation, increased mucus production, and pronounced gas trapping (10, 11). These changes lead to increased dyspnea, which in turn leads to a decreased lung function and quality of life, and an increased mortality (9, 12, 13).

GBD report recommended that vaccination against Haemophilus influenza type b should be offered that is largely based on reducing severe illness (e.g., lower respiratory tract infections requiring hospitalization) and death in patients with COPD (14, 15). The US Centers for Disease Control (CDC) has endorsed the pneumococcal vaccine because of its demonstrated ability to lower the occurrence of AECOPD and comorbidities of COPD (16). Notably, Non-typable Haemophilus influenzae (NTHi) and Moraxella catarrhalis (Mcat) have been identified as the predominant bacteria responsible for AECOPD (17–20). Furthermore, NTHi and Mcat often act as co-pathogens in COPD, thereby exacerbating issues related to antibiotic resistance and host immune response when co-infections occur (21, 22). In response to this challenge, an experimental adjuvant multicomponent vaccine has been developed to target NTHi and Mcat, aiming to enhance local airway immunity and pathogen-specific antibody production, thereby potentially reducing the frequency and severity of AECOPD episodes (23).

While several randomized controlled trials (RCTs) have investigated the efficacy and safety of NTHi-Mcat/NTHi vaccines in COPD patients, the findings have been inconclusive. Some RCTs reported positive outcomes, indicating that the vaccine is both safe and effective (24). Conversely, other RCTs have failed to demonstrate the efficacy of the vaccine in reducing AECOPD occurrence in COPD patients (25–28). In a recent Cochrane review, meta-analyses were conducted to evaluate the efficacy of the NTHi vaccine, focusing on adults with chronic bronchitis or COPD (29). This review was an update of a previous publication from 1998, which primarily aimed to assess the protective effects of the NTHi vaccine against recurrent acute episodes in patient with chronic bronchitis (30). Although the 2017 Cochrane review included studies involving adults with chronic bronchitis and COPD, it did not specifically focus on COPD subpopulations or perform robust subgroup analyses, despite incorporating three trials with COPD patients (24, 31, 32). Consequently, there remains a gap in the evidence, as no meta-analysis has specifically evaluated the efficacy of the NTHi vaccine in COPD patients. Our study aims to fill this gap by providing a focused analysis of this population.

Given the evolving landscape of studies on this topic, including recent evidence published in the last few years (23, 26–28), we sought to conduct a comprehensive systematic review and meta-analysis to evaluate the efficacy and safety of NTHi-Mcat/NTHi vaccines in COPD patients. Additionally, we sought to explore potential effect differences by drug delivery methods (e.g., oral vs. intramuscular) and vaccine composition (e.g., NTHi alone vs. NTHi-Mcat). The objective of this study is to offer valuable insight for future studies and guideline development in this area.

## Methods

Reporting of the systematic review was guided by the Preferred Reporting Items for Systematic Reviews and Meta-Analyses (PRISMA) guidelines (33). The study was registered in PROSPERO (ID: CRD42023381488).

### Search strategy

Regarding the search strategy, articles were searched in electronic databases, including PubMed, Cochrane Central Register of Controlled Trials (CENTRAL) databases, Embase (OVID), and Medline (OVID) from inception to October 2024 with “chronic obstructive pulmonary disease (COPD),” “vaccine, “Non-typeable Haemophilus influenzae (NTHi),” and “randomized controlled trial (RCT)” as search terms were retrieved without any restriction of date or language. The reference list of all selected articles was independently screened to identify additional studies. Details of the search strategy are shown in Supplementary Table S1.

### Study selection inclusion and exclusion criteria

Studies were included if the following criteria were met: (1) participants diagnosed with COPD or demonstrating immunological characteristics consistent with those of the COPD population, acknowledging the early onset of immune system alterations in smokers prior to COPD manifestation (34–36); (2) adherence to a RCT study design; (3) inclusion of multiple study groups, with at least one group receiving a vaccine containing NTHi or a combination of NTHi and Mcat (NTHi-Mcat); (4) evaluation of the safety and efficacy of the NTHi-Mcat vaccine in COPD patients. Primary outcomes of interest encompassed the incidence of AECOPD, all-cause mortality, hospitalization rates, and a comprehensive array adverse event, spanning serious adverse events, general reactions, and system-specific events affecting the gastrointestinal, respiratory, musculoskeletal, nervous, renal, cardiac, and immune systems. Exclusion criteria were: (1) literature reviews, quasi-randomized or cluster-randomized trials, editorials, conference abstracts, correspondence, and case reports; (2) duplicate publications; (3) studies focused on basic or animal research; (4) investigations focusing solely on reactogenicity or the humoral and cellular immunogenicity of the NTHi-Mcat vaccine; (5) studies lacking adequate data for analysis. If multiple publications from the same trial were identified, we extracted and retained all available data. For outcomes with overlapping populations reported across multiple publications, we prioritized the results with the longest follow-up duration or the largest sample size to ensure the most comprehensive and reliable data were included.

After deleting duplicate studies, two reviewers independently reviewed the title and abstract according to the inclusion criteria. Subsequently, reviewers continued to screen the full text according to the inclusion criteria, and finally the study would be included in the review. Discrepancies in the selection process were resolved through discussion or, if necessary, by consultation with a third reviewer.

### Data extraction and quality assessment

Two reviewers independently extracted data and assessed the risk of bias using a standardized data abstraction form. The following information was extracted: (1) study characteristics: first author, publication year, phase, center, blinding, study duration, and follow-up; (2) participant characteristics: mean age, gender, sample size, and region; (3) intervention and comparator information: components of the vaccine, vaccine types, and delivery methods; and (4) outcomes: effect size, and 95% confidence intervals (CI). In case of uncertainty, the reviewers consulted each other. Discrepancies during the data extraction and risk of bias assessment were resolved through discussion or, if necessary, by consultation with a third reviewer.

The risk of bias was assessed using the RCT risk of bias assessment tool (RoB) as recommended in the Cochrane Handbook of Systematic Reviews (37). The evaluation included the following: random sequence generation, allocation concealment, whether to use blinding to participants and personnel, whether to use blinding to the measurers of the study outcome, whether the outcome data were complete, whether to selectively report the study results and other sources of bias. Each domain was rated as “uncertain,” “low risk,” or “high risk.”

### Statistical analyses

Meta-analyses were performed using R version 4.2.2. The impact magnitude of the measured data was expressed as relative risks (RRs) and 95% CI. Depending on the *I^2^* and *p* values, the data were combined using either a random-effects or fixed-effects model. When the *I^2^* statistic was greater than 50% or the *p*-value was less than 0.05, indicating significant heterogeneity, the random-effects model was used to compute the pooled effect sizes (38). Otherwise, the fixed-effects model was applied. The restricted maximum-likelihood estimator of *τ^2^* was used to assess the between-study heterogeneity (39). The Hartung–Knapp method was used for adjustment of the estimates and confidence intervals (CIs) for random-effects model (40).

To further evaluate heterogeneity, subgroup analyses were conducted to evaluate the results of standardized meta-analysis. Subgroup analyses were conducted based on intervention characteristics (e.g., drug types, methods of administration), region (Europe vs. Australia vs. mixed), sample size (≥50 vs. <50), and study design (multicenter vs. single center). To comprehensively assess the relative impact of each study, sequential removal of each study for sensitivity analysis was performed. Funnel plot, the Egger’s test (41) and Begg’s test (42) were used to assess for publication bias if more than 10 studies were included in the primary outcomes.

## Results

**Figure 1:**
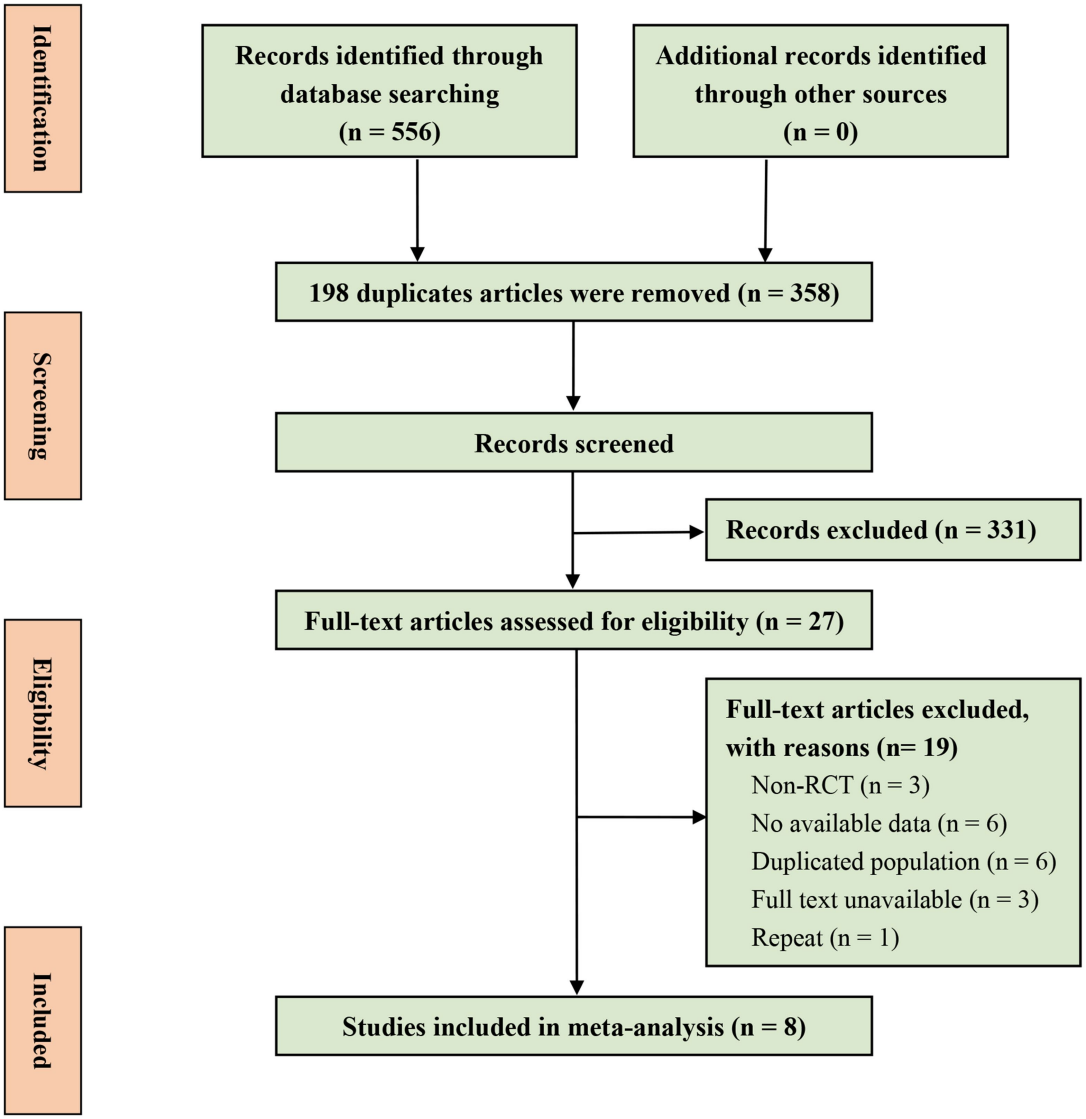
The flow chart of the study selection.

A total of 556 records were initially retrieved through the search strategy, and after removing 198 duplicates, 358 articles were included for further screening. These articles were assessed based on title and abstract, resulting in 27 full-text articles being examined for eligibility. After excluding 19 records (details are shown in Supplementary Table S2), eight studies were ultimately included in the analysis (Figure 1).

### Characteristics of the studies

Eight eligible RCTs included a total of 1,574 participants, with sample sizes ranging from 33 to 606. The characteristics of the studies are shown in Table 1 and Supplementary Table S3. The majority of the studies were conducted in Europe (*n* = 3) (23, 27, 28), followed by two studies from Australia (31, 32), one study from the USA and Australia (24), and one study (25) with undisclosed participant origin (Table 1). Drugs were administered orally (24, 25, 31, 32) or via intramuscular injection (23, 26–28). The administration frequency varied, with some studies administering the intervention twice (23, 26–28) and others three times (31, 32); in one study the administration frequency was not specified. One study has two control groups (32), and two studies have two intervention groups (23, 28).

**Table 1:** Characteristics of randomized trials included in systematic review and meta-analysis.

First author	Year	Phase	Multicenter	Blinding	Study duration	Sample size		Country	Follow-up (Months)	Age (Mean)	Men (%)	Intervention		Control		Funding
						Total	I/C			I/C	I/C	Vaccine	Delivery methods	Drug	Delivery methods	
Andreas S. (26)	2022	Phase 2b trial	Yes	Observer-blinded	2017/11–2020/03	606	304/302	Belgium, Canada, France, Germany, Italy, Spain, UK, and USA.	15	65.7/66.3	60.5/58.6	NTHi–Mcat vaccine	Intramuscular injection	Phosphate-buffered saline	Intramuscular injection	GlaxoSmithKline Biologicals SA.
Clancy R. (32)	1985	NR	No	Double-blind	Winter period in 1983	34	17/17	Australia	3	64.7/62.0	82.4/94.1	NTHi vaccine	Oral	25 mg sodium tauroglycocholate	Oral	GlaxoSmithKline Biologicals SA
						33	17/16	Australia	3	64.7/65.5	82.4/62.5	NTHi vaccine	Oral	Enteric-coated glucose tablet	Oral	Ciba-Geigy (Australia)
Clancy R. L. (31)	2016	NR	Yes	Double-blind	Winter of 2011	320	160/160	Australia	9	71.2/67.9	66.9/58.1	NTHi vaccine	Oral	NR	Oral	Bioxyne Ltd
De Smedt P. (28)	2021	Phase 1 trial	Yes	Observer-blinded	2015/8–2017/4	55	27/28	Belgium	48	59.7/58.2	55.6/67.9	NTHi–Mcat vaccine	Intramuscular injection	Saline solution	Intramuscular injection	GlaxoSmithKline Biologicals SA
						54	26/28	Belgium	48	59.0/58.2	53.8/67.9	NTHi–Mcat vaccine	Intramuscular injection	Saline solution	Intramuscular injection	GlaxoSmithKline Biologicals SA
Tandon M. K. (24)	2010	Phase 2 trial	Yes	Double-blind	2023/10/6	38	18/20	USA and AUS	4	69.5/67.3	83.3/70.0	NTHi vaccine	Oral	Enteric-coated placebo tablet	Oral	Hunter Immunology Ltd
Philips M. (25)	2007	NR	No	Double-blind	NR	140	68/72	Australia	NR	NR/NR	NR/NR	NTHi vaccine	Oral	NR	NR	NR
Van Damme P. (23)	2019	Phase 1 trial	Yes	Observer-blinded	2015/8–2017/3	75	31/44	Belgium	12	58.9/47.2	58.1/52.3	NTHi–Mcat vaccine	Intramuscular injection	Saline solution	Intramuscular injection	GlaxoSmithKline Biologicals SA
						74	30/44	Belgium	12	58.5/47.2	56.7/52.4	NTHi–Mcat vaccine	Intramuscular injection	Saline solution	Intramuscular injection	GlaxoSmithKline Biologicals SA
Wilkinson T. M. A. (27)	2019	Phase 2 trial	Yes	Observer-blinded	2014/7/8–2017/4/19	145	73/72	Sweden and UK	15	67.0/66.8	53.4/50.0	NTHi vaccine	Intramuscular injection	Saline placebo	Intramuscular injection	GlaxoSmithKline Biologicals SA

NTHi–Mcat vaccine, non-typeable Haemophilus influenzae-Moraxella catarrhalis vaccine; NTHi vaccine, non-typeable Haemophilus influenzae vaccine; NR, not reported; I, intervention; C, control.

### Risk of bias assessment

The RoB assessment (Supplementary Figure S1) showed that high risk of bias was primarily attributed to reporting bias. Specifically, two studies (24, 25) had incomplete reporting of adverse reaction outcomes, possibly due to a different focus of the study as it was not possible to report the important outcome measures consistently to all studies.

### The efficacy of non-typeable Haemophilus influenzae vaccine in COPD

**Figure 2:**
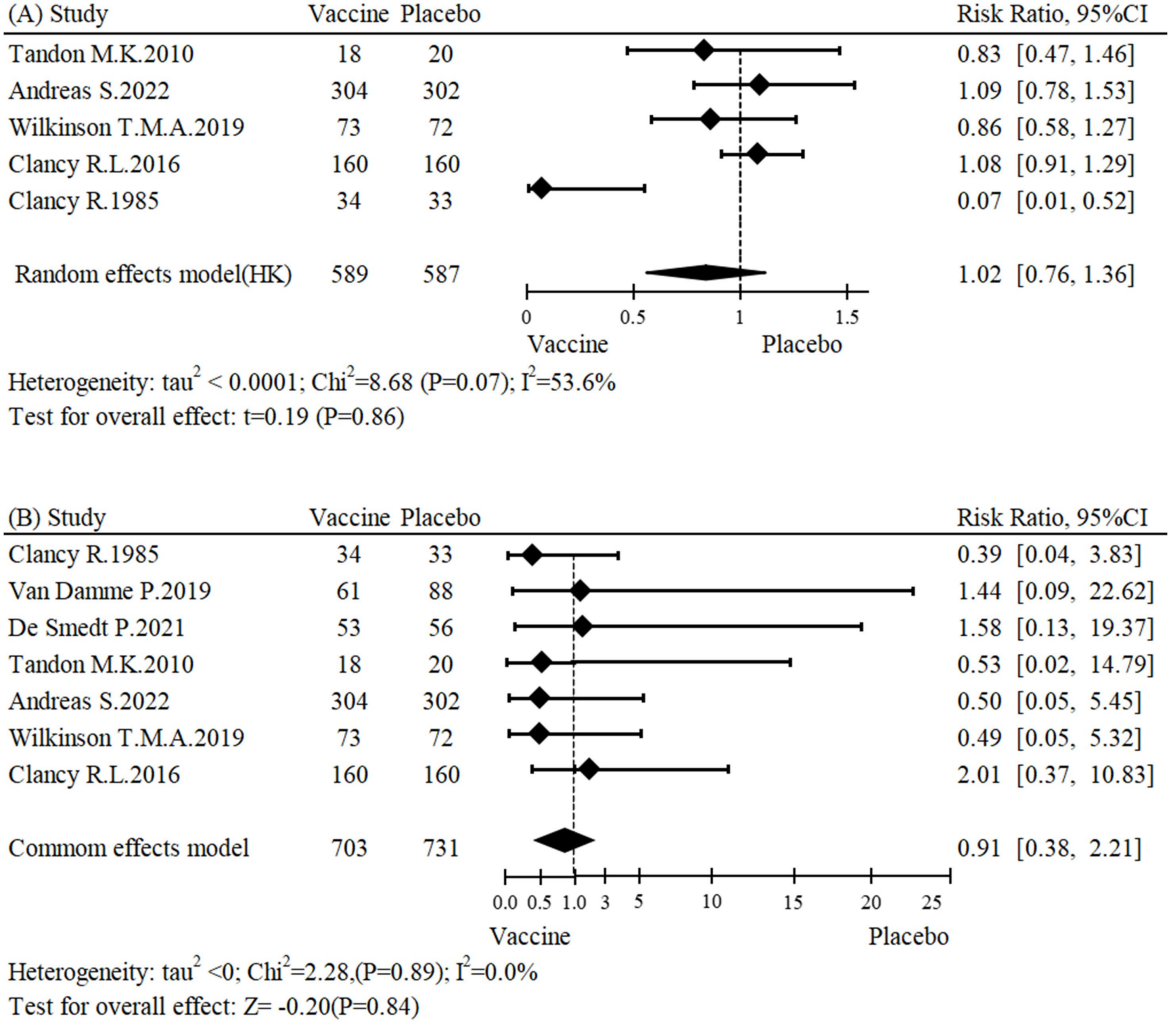
Forest plot for **(A)** incidence of AECOPD and **(B)** all-cause mortality.

This meta-analysis included five studies (24, 26, 27, 31, 32) with a total of 1,176 participants. The results showed that the NTHi-Mcat/NTHi vaccine did not have a significant effect on the risk of AECOPD occurrence (RR: 1.02, 95% CI: 0.76 to 1.36; Figure 2). Sensitivity analysis was conducted and the stability and reliability of the findings were confirmed (Supplementary Figure S2). The research findings presented in Table 2 suggest that the NTHi-Mcat/NTHi vaccine does not increase the risk of AECOPD in multicenter studies (*n* = 4, RR: 1.03, 95% CI: 0.90 to 1.19). Additionally, subgroup analyses showed no significant differences in AECOPD risk based on region (*p* = 0.69), sample size (*p* = 0.46), intervention types (*p* = 0.67), and drug delivery methods (*p* = 0.74).

**Table 2:** Subgroup analyses of efficacy and safety of non-typeable Haemophilus influenzae vaccine in chronic obstructive pulmonary disease.

Subgroups	No. of studies	No. of patients	Effect size	Heterogeneity		*p* interaction
			RR (95%CI)	*I^2^* (%)	*p* value	
AECOPD						
Region						
Europe	2	751	0.98 (0.76,1.27)	0	0.37	0.69
Australia	2	387	1.06 (0.89,1.26)	86	<0.01	
Mixed	1	38	0.83 (0.47,1.46)	─	─	
Center						
Multicenter	4	1,109	1.03 (0.90,1.19)	0	0.62	<0.01
Single center	1	67	0.07 (0.01,0.52)	─	─	
Sample sizes						
≥ 50	4	1,138	1.03 (0.90,1.19)	63	0.04	0.46
< 50	1	38	0.83 (0.47,1.46)	─	─	
Intervention types						
NTHi vaccine	4	570	1.01 (0.86,1.17)	65	0.04	0.67
NTHi–Mcat vaccine	1	606	1.09 (0.78,1.53)	─	─	
Drug delivery methods						
Intramuscular injection	2	751	0.98 (0.76,1.27)	0	0.37	0.74
Oral	3	425	1.04 (0.88,1.22)	74	0.02	
All-cause mortality						
Region						
Europe	4	1,009	0.82 (0.24,2.86)	0	0.86	0.89
Australia	2	387	1.13 (0.29,4.37)	22	0.26	
Mixed	1	38	0.53 (0.02,14.80)	─	─	
Center						
Multicenter	6	1,367	1.06 (0.41,2.77)	0	0.89	0.43
Single center	1	67	0.39 (0.04,3.83)	─	─	
Sample size						
≥ 50	6	1,396	0.95 (0.38,2.38)	0	0.83	0.74
< 50	1	38	0.53 (0.02,14.80)	─	─	
Drug of interventions						
NTHi vaccine	4	570	0.87 (0.29,2.63)	0	0.63	0.88
NTHi–Mcat vaccine	3	864	1.00 (0.23,4.32)	0	0.77	
Drug delivery methods						
Intramuscular injection	4	1,009	0.82 (0.24,2.86)	0	0.86	0.82
Oral	3	425	1.01 (0.29,3.56)	0	0.48	
Hospitalization						
Region						
Europe	0	-				
Australia	3	527	1.29 (1.00,1.68)	91	<0.01	0.06
Mixed	1	38	1.23 (0.95,1.58)	-	-	
Center						
Single-center	2	207	0.30 (0.15,0.59)	7	0.3	<0.01
Multicenter	2	358	1.54 (1.17,2.03)	81	0.02	
Sample sizes						
<50	1	38	0.42 (0.13,1.33)	-	-	
≥50	3	527	1.29 (1.00,1.68)	91	<0.01	0.06
Serious adverse events						
Region						
Europe	4	1,009	0.91 (0.73,1.12)	0	0.62	0.13
Australia	1	320	1.19 (0.90,1.58)	─	─	
Drug of Interventions						
NTHi vaccine	2	465	1.13 (0.87,1.46)	0	0.37	0.23
NTHi–Mcat vaccine	3	864	0.91 (0.72,1.15)	0	0.41	
Drug delivery methods						
Intramuscular injection	4	1,009	0.91 (0.73,1.12)	0	0.62	0.13
Oral	1	320	1.19 (0.90,1.58)	─	─	
Grade 3 serious adverse events						
Region						
Europe	3	900	1.35 (0.92,1.98)	0	0.39	0.43
Australia	1	320	1.11 (0.81,1.52)	─	─	
Drug of interventions						
NTHi vaccine	2	465	1.07 (0.79,1.45)	0	0.41	0.20
NTHi–Mcat vaccine	2	755	1.49 (0.99,2.23)	0	0.68	
Drug delivery methods						
Intramuscular injection	3	900	1.35 (0.92,1.98)	0	0.39	0.43
Oral	1	320	1.11 (0.81,1.52)	─	─	
pIMDs						
Drug of interventions						
NTHi vaccine	1	145	3.95 (0.18,85.99)	─	─	0.67
NTHi–Mcat vaccine	3	864	1.94 (0.65,5.74)	0	0.98	

AECOPD, Acute exacerbation of chronic obstructive pulmonary disease; NTHi–Mcat vaccine, non-typeable Haemophilus influenzae-Moraxella catarrhalis vaccine; NTHi vaccine, non-typeable Haemophilus influenzae vaccine; pIMDs, potential immune-mediated diseases.

A meta-analysis of seven studies (23, 24, 26–28, 31, 32), encompassing a total of 1,434 participants, assessed the impact of the NTHi-Mcat/NTHi vaccine on all-cause mortality. The findings showed that the vaccine did not have a significant effect on the risk of all-cause mortality (RR: 0.91, 95% CI: 0.38 to 2.21; Figure 2). Furthermore, sensitivity analysis confirmed the stability of the results (Supplementary Figure S3). Subgroup analyses were conducted to investigate potential sources of heterogeneity, including region (*p* = 0.89), center (*p* = 0.43), sample size (*p* = 0.74), intervention types (*p* = 0.88), and drug delivery methods (*p* = 0.82). None of these factors were found to be significantly associated with heterogeneity (Table 2).

### The safety of non-typeable Haemophilus influenzae vaccine in COPD

**Figure 3:**
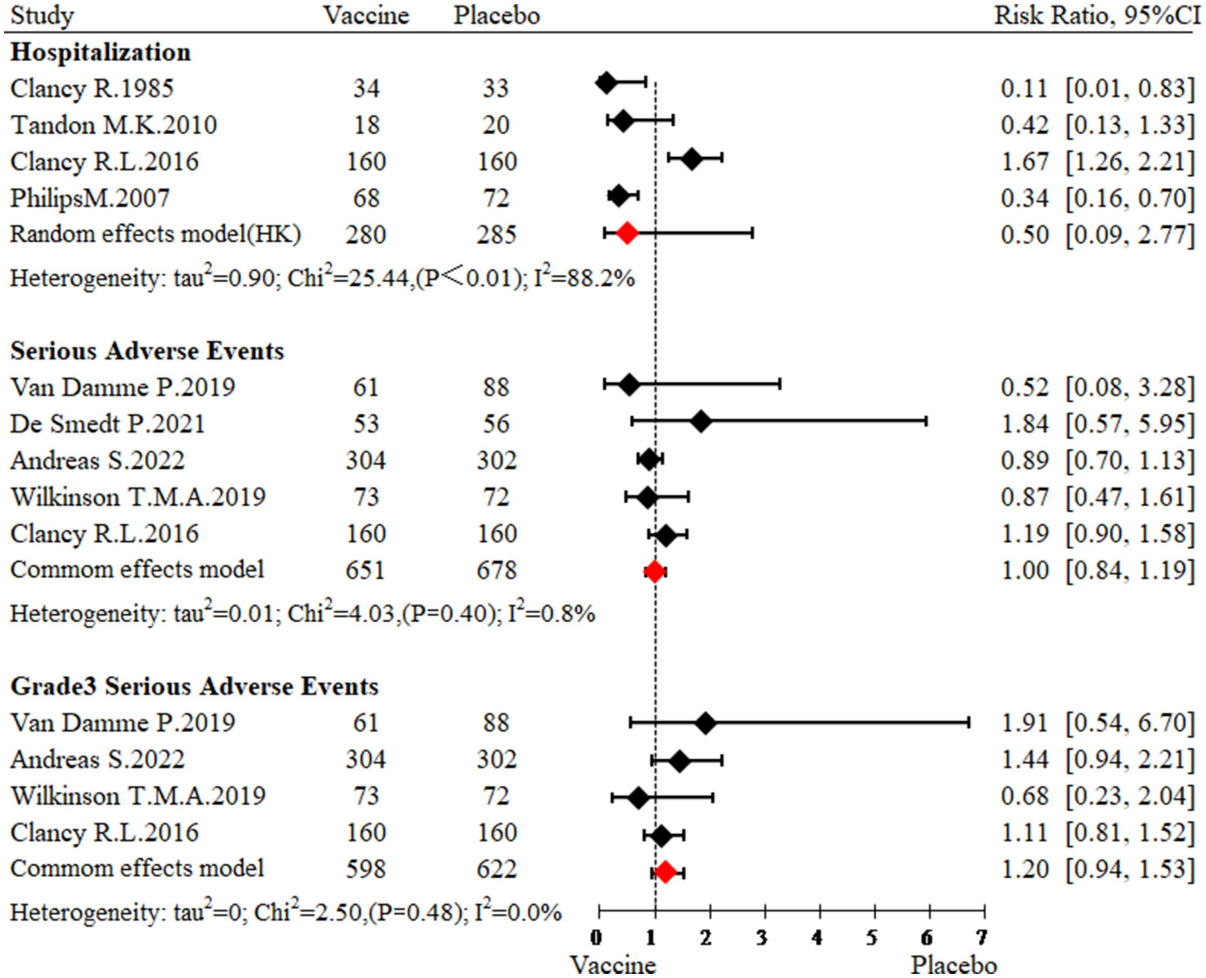
Meta-analysis for safety outcomes.

The meta-analysis of five studies (23, 26–28, 31) involving 565 participants, showed that the NTHi-Mcat/NTHi vaccine did not affect the incidence of hospitalization (RR: 0.50, 95% CI: 0.09 to 2.77; Figure 3). Sensitivity analyses indicated that the results were stable (Supplementary Figure S4). Moreover, subgroup analysis revealed that the NTHi-Mcat/NTHi vaccine was not associated with a risk of hospitalization in multicenter studies (*n* = 2, RR: 1.54, 95% CI: 1.17 to 2.03). However, in single-center studies (*n* = 2), the NTHi-Mcat/NTHi vaccine significantly decreased the risk of hospitalization (RR: 0.30, 95% CI: 0.15 to 0.59). Furthermore, subgroup analyses did not indicate region (*p* = 0.06) and sample size (*p* = 0.06) to be significant sources of heterogeneity (Table 2).

In addition to evaluating the efficacy of NTHi-Mcat/NTHi vaccines in COPD patients, the safety profile was also comprehensively analyzed. This analysis encompassed a wide range of adverse events, including serious adverse events, general events, and events that were specific to different bodily systems, such as gastrointestinal, respiratory, musculoskeletal, nervous, renal, cardiac, and immune system disorders (Supplementary Table S4). To ensure a robust assessment, a meta-analysis of these safety outcomes was conducted when at least three studies reported on a specific event.

The meta-analysis of five studies (25, 27, 31, 32) involving 1,329 participants indicated that administration of the NTHi-Mcat/NTHi vaccine had no significant impact on the incidence of serious adverse events (RR: 1.00, 95% CI: 0.84 to 1.19; Figure 3). Subgroup analyses were performed to explore potential sources of heterogeneity, including region (*p* = 0.13), intervention types (*p* = 0.23), and drug delivery methods (*p* = 0.13), but no significant association was found (Table 2).

Four studies (23, 26, 27, 31), including a total of 1,220 participants, were included in this meta-analysis. The analysis indicated that the NTHi-Mcat/NTHi vaccine did not increase risk of grade 3 serious adverse events (RR: 1.20, 95% CI: 0.93 to 1.53; Figure 3). Furthermore, subgroup analyses were conducted to explore potential sources of heterogeneity, but no significant association was found between region (*p* = 0.43), intervention types (*p* = 0.20), drug delivery methods (*p* = 0.43), and heterogeneity (Table 2).

The findings from the meta-analysis regarding other safety outcomes are presented in Table 3. While there were some differences in general adverse events between the NTHi-Mcat/NTHi vaccine group and the control group, no significant differences in adverse events were found regardless of timing (at any time, after the first dose, or after the second dose). However, compared to the control group, individuals in the NTHi-Mcat/NTHi vaccine group had a higher likelihood of experiencing pain and swelling at any time (pain: *n* = 3, RR: 5.33, 95% CI: 1.98 to 14.33; swelling: *n* = 3, RR: 12.15, 95% CI: 4.67 to 31.67), after the first dose (pain: *n* = 3, RR: 7.72, 95% CI: 5.52 to 10.79; swelling: *n* = 3, RR: 6.66, 95% CI: 1.88 to 33.76), and after the second dose (pain: *n* = 3, RR: 7.96, 95% CI: 1.98 to 14.33; swelling: *n* = 3, RR: 16.10, 95% CI: 3.78 to 68.60). Moreover, individuals in the NTHi-Mcat/NTHi vaccine group were more likely to experience redness after both the first dose (*n* = 3, RR: 12.74, 95% CI: 3.48 to 46.59) and the second dose (*n* = 3, RR: 11.55, 95% CI: 3.90 to 34.22). Additionally, individuals in the NTHi-Mcat/NTHi vaccine group had a higher risk of developing headaches (any time: *n* = 3, RR: 1.20, 95% CI: 1.00 to 1.43; after the first dose: *n* = 3, RR: 1.30, 95% CI: 1.03 to 1.64), erythema (any time: *n* = 3, RR: 15.38, 95% CI: 5.64 to 41.92), and fever (after the second dose: *n* = 3, RR: 2.33, 95% CI: 1.24 to 4.38). Notably, the increased risk of injection-site-related events (pain, swelling, redness) is consistent with many vaccine studies.

**Table 3:** The safety of non-typeable Haemophilus influenzae vaccine in chronic obstructive pulmonary disease.

General adverse events	Sample size	No. of event	No. of studies	Rate ratio, 95%CI	*p* value	Heterogeneity		
						*I^2^*	τ^2^	*p* value
Any								
Fatigue	894	502	3	1.28 (0.59, 2.78)	0.31	74.1	0.08	0.02
Fever	1,003	99	3	1.38 (0.94, 2.01)	0.10	21.0	0.04	0.28
Pain	900	386	3	5.33 (1.98, 14.33)	0.02	74.5	0.12	0.02
Headache	894	330	3	1.20 (1.00, 1.43)	0.05	0.0	0.0	0.64
Swelling	900	55	3	12.15 (4.67, 31.61)	<0.01	0.0	0.0	0.96
pIMDs	1,009	13	4	2.10 (0.75, 5.84)	0.16	0.0	0.0	0.98
Erythema	900	75	3	15.38 (5.64,41.92)	<0.01	0.0	0.0	0.47
Nasopharyngitis	900	71	3	0.95 (0.61, 1.49)	0.84	0.0	0.0	0.51
Oropharyngeal pain	900	16	3	0.90 (0.35,2.34)	0.83	0.0	0.0	0.87
Gastroenteritis	938	7	4	0.84 (0.22,3.22)	0.79	0.0	0.0	0.77
Gastrointestinal disorders	900	163	3	0.96 (0.73,1.25)	0.74	0.0	0.01	0.54
Pneumonia	900	25	3	0.50 (0.22,1.16)	0.11	18.2	0.27	0.29
Rib fracture	900	3	3	2.07 (0.37,11.67)	0.41	0.0	0.0	1.00
Upper respiratory tract infection	822	23	3	1.21 (0.54,2.68)	0.65	0.0	0.0	0.88
Other adverse events^*^	900	751	3	1.27 (0.86, 1.87)	0.12	80.5	0.02	0.01
After dose 1								
Redness	877	33	3	12.74 (3.48, 46.59)	<0.01	0.0	0.0	0.80
Fatigue	877	398	3	0.95 (0.82, 1.09)	0.44	0.0	0.0	0.86
Fever	877	59	3	0.92 (0.56, 1.50)	0.73	34.5	0.0	0.22
Pain	877	280	3	7.72 (5.52, 10.79)	<0.01	22.9	0.04	0.27
Headache	877	232	3	1.30 (1.03, 1.64)	0.03	0.0	0.0	0.91
Swelling	877	23	3	6.66 (2.16, 20.53)	<0.01	0.0	0.0	0.99
Gastrointestinal symptoms	877	133	3	0.93 (0.68, 1.27)	0.63	0.0	0.0	0.75
After dose 2								
Redness	823	61	3	11.55 (3.90, 34.22)	<0.01	6.8	0.0	0.34
Fatigue	823	341	3	1.72 (0.51, 5.83)	0.20	83.3	0.21	<0.01
Fever	823	48	3	2.33 (1.24, 4.38)	0.01	39.9	0.36	0.19
Pain	823	294	3	7.96 (1.88, 33.76)	0.03	78.6	0.27	0.01
Headache	823	189	3	1.77 (0.34, 9.07)	0.27	67.2	0.26	0.05
Swelling	823	44	3	16.10 (3.78. 68.60)	<0.01	0.0	0.0	0.48
Gastrointestinal symptoms	823	102	3	1.40 (0.96, 2.03)	0.08	47.4	0.19	0.15

^*^Not Including Serious adverse events; pIMDs, potential immune-mediated diseases.

Based on the evidence available, the NTHi-Mcat/NTHi vaccine appears to be safe for use in individuals with COPD. No significant differences were observed in the occurrence of major adverse events between vaccine and placebo groups. However, mild adverse events, such as fatigue, headaches, myalgia, and fever were reported at similar rates in both groups, following both the initial and subsequent doses of the vaccine. These findings were further supported by sensitivity analyses, which enhanced the reliability and consistency of the results obtained (Supplementary Figures S5–S7).

## Discussion

This study is a comprehensive systematic review and meta-analysis conducted to assess the efficacy and safety of NTHi-Mcat/NTHi vaccines in COPD patients. Our study findings show no statistically significant differences in adverse events between the NTHi-Mcat/NTHi vaccine group and the control group at any time point, post-first dose, or post-second dose, except for six commonly reported events: pain, swelling, redness, headaches, erythema, and fever. Furthermore, our evidence supports that NTHi-Mcat/NTHi vaccines do not reduce the risk of AECOPD or overall mortality. These findings are significant for clinical practice and future research in this area.

In previous studies (29, 43), it has been reported that vaccination has no significant effect on the number of AECOPD, which is consistent with the findings obtained in our study. However, our study expanded previous results in two critical aspects. First, a larger number of studies was included, totaling 8 articles with a collective sample size of 2,180 participants. The larger sample size encompassed a diverse range of participants, including individuals from different regions, ethnicities, and age groups, thereby enhancing the reliability and generalizability of our results. Consequently, to explore the potential sources of heterogeneity, subgroup analyses were conducted based on various factors, such as regions, centers, intervention types, sample size, and drug delivery methods. Moreover, a comprehensive investigation into the safety of vaccination was conducted, taking into account general adverse events as well as events specific to different bodily systems, including gastrointestinal, respiratory, musculoskeletal, nervous, renal, cardiac, and immune system disorders.

The mechanism of action underlying the efficacy of NTHi-Mcat/NTHi vaccines in patients with COPD likely involves the stimulation of a robust immune response against these pathogens (44). This immune response is characterized by the generation of pathogen-specific antibodies (45) and the activation of cellular immunity (27). Additionally, the vaccines exhibit immunomodulatory effects within the airway microenvironment, thereby contributing to enhanced respiratory function and reduced exacerbations in COPD patients. Nevertheless, our research findings indicate that NTHi-Mcat/NTHi vaccines do not significantly impact the risk of AECOPD or all-cause mortality in COPD patients. This lack of effect may be attributed to the marked genetic heterogeneity of NTHi, including its phase-variable genes, as well as the high genetic variability of NTHi or differences in patients’ baseline immunity. These factors may all potentially reduce the efficacy of the vaccine (46). Further investigations are warranted to elucidate the mechanisms underlying the effects of these vaccines and to explore biomarkers that can predict vaccine responses, thereby helping to identify specific patient subgroups who may benefit from the vaccines.

The overall heterogeneity in this study was low (*I^2^* < 50%), suggesting that heterogeneity had a limited impact on the results. To further explore the sources of heterogeneity and its potential influence, subgroup and sensitivity analyses were conducted. The heterogeneity observed in hospitalization outcomes primarily originated from one single-center study (32). This may be attributed to limitations in sample selection, research methods, or regional differences inherent in single-center studies. Potential explanations include variations in the standard of care, disease severity, or differing definitions of ‘hospitalization’ across study settings.” Nevertheless, this study effectively identified and controlled the main source of heterogeneity through sensitivity analysis, significantly enhancing the robustness of the results. Future research could further validate and optimize the findings by expanding sample sizes and incorporating more high-quality multicenter studies, thereby reducing the potential impact of heterogeneity and improving the generalizability of the conclusions.

Although our study did not show a significant impact of the NTHi-Mcat/NTHi vaccine on the risk of AECOPD and all-cause mortality in COPD patients, it is important to note that this does not negate the potential efficacy of the vaccine in a specific population. Future research should focus on exploring the efficacy of the vaccine in subgroups, such as those with severe COPD or confirmed infections. The overall safety and efficacy of the NTHi-Mcat/NTHi vaccine still remain controversial, and further studies should consider individual differences and personalized vaccine strategies. To improve vaccine efficacy and minimize side effects, future research can delve into patient genotypes, immune status, and pathogen characteristics to develop more targeted vaccines. Personalized vaccine strategies may require the integration of genetic testing, immunological evaluation, and clinical data to provide tailored preventive measures for each patient. It is crucial to ensure the safety of vaccines to implement effective vaccination programs. Although serious adverse events have not been identified in current studies, continuous monitoring of the safety of newly developed vaccines is necessary. Future studies should focus on long-term safety monitoring and establish effective mechanisms for safety management to promptly assess and manage any potential side effects.

### Strengths and limitations

This is the first systematic review and meta-analysis to investigate the safety and efficacy of NTHi-Mcat/NTHi vaccines in COPD patients. However, this study has several limitations. First, the participants in our meta-analyses were predominantly from Europe and Australia, which limits the generalizability of our findings to other regions, such as Asia, Africa, or the Americas. Therefore, the results should be interpreted with caution when applied to other populations. Second, subgroup analyses were conducted and more than 5 studies were included to explore the sources of heterogeneity and the impact of factors on the efficacy of NTHi-Mcat/NTHi vaccines in COPD patients based on vaccine composition, administration route, and dosage. However, the number of studies included in our meta-analyses was fewer than ten, and the sample sizes within subgroups were limited. These factors may reduce the statistical power of the subgroup analyses and limit the generalizability of the findings. Third, variations in participant age and gender, definitions of COPD exacerbations or severity, and vaccine dosage may influence the efficacy of NTHi-Mcat/NTHi vaccines. However, due to limited data availability, we were unable to conduct analyses to explore the impact of these factors on vaccine efficacy in COPD patients.

## Conclusion

In summary, our findings indicate that the administration of NTHi-Mcat/NTHi vaccines in COPD patients did not result in any significant adverse events. However, no significant reduction in the risk of AECOPD or overall mortality was observed when using these vaccines. To further validate these results, future studies should prioritize larger sample sizes and longer follow-up periods, particularly in the Asian population, to enhance the generalizability of the findings. Additionally, we recommend conducting multinational RCTs focusing on specific subgroups, such as patients with severe COPD, frequent exacerbations, or confirmed NTHi colonization. Furthermore, investigating immunological correlates of protection—such as baseline antibody titers or immune signatures—may help identify subgroups more likely to benefit from vaccination, providing a deeper understanding of vaccine efficacy in this population.

## Data Availability

The original contributions presented in the study are included in the article/Supplementary material, further inquiries can be directed to the corresponding author.
