# Supplementary material for: The safety and efficacy of non-typeable Haemophilus influenzae and Moraxella catarrhalis vaccine in chronic obstructive pulmonary disease: a systematic review and meta-analysis of randomized controlled trials
Source: Front Med (Lausanne). 2025 Apr 4;12:1572726. doi: 10.3389/fmed.2025.1572726 (PMC12006075; doi:10.3389/fmed.2025.1572726)

## Table S1. Search strategy

| Embase | |
| --- | --- |
| 1 | exp haemophilus/ |
| 2 | ("Hemophilus" or "haemophilus" or "haemophilus isolation" or "Mycobacterium influenzae" or "Haemophilus meningitidis" or "Influenza-bacillus" or "Hemophilus influenzae" or "Coccobacillus pfeifferi" or "bacterium influenzae" or "h. influenzae" or "haemophilus influenza" or "hemophilus influenza extract" or "influenza bacillus" or "pfeiffer bacillus" or "haemophilus influenzae" or "non typeable haemophilus influenzae" or "Non-typeable Haemophilus influenza" or "NTHi-Mcat" or "nontypeable Haemophilus influenzae ").af. |
| 3 | exp immunization/ |
| 4 | exp immunotherapy/ |
| 5 | exp Vaccine/ |
| 6 | ("immunotherapy" or "Vaccines" or "Vaccine" or "Sensitization, Immunologic" or "Immunizations" or "Immunotherapies" or "Immunological Sensitizations" or "Immunological Sensitization" or "Sensitization, Immunological" or "Immunostimulation" or "Immunologic Stimulation" or "Sensitizations, Immunological" or "biologic response modifier therapy" or "biological response modifier therapy" or "brm therapy" or "immune therapy" or "immunisation" or "immunisation efficiency" or "immunisation prophylaxis" or "immunisation schedule" or "immunization" or "immunization efficiency" or "immunization prophylaxis" or "immunization schedule" or "immunogenic therapy" or "immunoglobulin therapy" or "immunologic sensitization" or "Immunological Stimulation" or "Immunological Stimulations" or "immunological therapy" or "immunological treatment" or "immunomodulant therapy" or "immunomodulary therapy" or "immunomodulating therapy" or "immunomodulation therapy" or "immunomodulative therapy" or "immunomodulator therapy" or "immunomodulatory intervention" or "immunomodulatory therapy" or "immunomoduling therapy" or "immunomodurating therapy" or "immunostimulation therapy" or "oral immunisation" or "oral immunization" or "reimmunisation" or "reimmunization" or "standard immunization program" or "Stimulation, Immunologic" or "Stimulation, Immunological" or "Stimulations, Immunological" or "Variolation" or "Variolations").ab,ti. |
| 7 | 3 or 4 or 5 or 6 |
| 8 | exp Haemophilus Vaccines/ |
| 9 | ("Non-typeable Haemophilus influenzae-Moraxella catarrhalis vaccine" or "Vaccines, Haemophilus" or "Hemophilus Vaccines" or "Vaccines, Hemophilus" or "Haemophilus influenzae Vaccines" or "Vaccines, Haemophilus influenzae" or "Haemophilus Vaccine" or "Non-typeable Haemophilus influenzae protein vaccine" or "inactivated nontypeable Haemophilus influenzae " or "non-typeable Haemophilus influenzae-Moraxella catarrhalis (NTHi-Mcat) vaccine" or "NTHi-Mcat vaccine" or "haemophilus vaccines" or "vaccine, haemophilus" or "hemophilus vaccine" or "hemophilus influenzae vaccine" or "hemophilus influenzae virus vaccine" or "Haemophilus influenzae vaccine").ab,ti. |
| 10 | exp Pulmonary Disease, Chronic Obstructive/ |
| 11 | ("Chronic Obstructive Lung Disease" or "Chronic Obstructive Pulmonary Diseases" or "COAD" or "COPD" or "Chronic Obstructive Airway Disease" or "Chronic Obstructive Pulmonary Disease" or "Airflow Obstruction, Chronic" or "Airflow Obstructions, Chronic" or "Chronic Airflow Obstructions" or "Chronic Airflow Obstruction" or "pulmonary disease, chronic obstructive" or "chronic airway obstruction" or "chronic obstructive bronchopulmonary disease" or "chronic obstructive lung disorder" or "chronic obstructive pulmonary disorder" or "chronic obstructive respiratory disease" or "chronic pulmonary obstructive disease" or "chronic pulmonary obstructive disorder" or "lung chronic obstructive disease" or "lung disease, chronic obstructive" or "obstructive chronic bronchitis" or "obstructive chronic lung disease" or "obstructive chronic pulmonary disease" or "obstructive lung disease, chronic" or "pulmonary disorder, chronic obstructive").ab,ti. |
| 12 | 10 or 11 |
| 13 | exp nontypeable Haemophilus influenzae/ |
| 14 | 1 or 2 or 13 |
| 15 | 7 and 14 |
| 16 | exp Haemophilus influenzae vaccine/ |
| 17 | exp Haemophilus vaccine/ |
| 18 | 8 or 9 or 16 or 17 |
| Ovid MEDLINE(R) and Epub Ahead of Print, In-Process, In-Data-Review & Other Non-Indexed Citations, Daily and Versions <1946 to November 11, 2022> | |
| 1 | exp haemophilus/ |
| 2 | ("Hemophilus" or "haemophilus" or "haemophilus isolation" or "Mycobacterium influenzae" or "Haemophilus meningitidis" or "Influenza-bacillus" or "Hemophilus influenzae" or "Coccobacillus pfeifferi" or "bacterium influenzae" or "h. influenzae" or "haemophilus influenza" or "hemophilus influenza extract" or "influenza bacillus" or "pfeiffer bacillus" or "haemophilus influenzae" or "non typeable haemophilus influenzae" or "Non-typeable Haemophilus influenza" or "NTHi-Mcat" or "nontypeable Haemophilus influenzae ").af. |
| 3 | 1 or 2 |
| 4 | exp immunization/ |
| 5 | exp immunotherapy/ |
| 6 | exp Vaccine/ |
| 7 | ("immunotherapy" or "Vaccines" or "Vaccine" or "Sensitization, Immunologic" or "Immunizations" or "Immunotherapies" or "Immunological Sensitizations" or "Immunological Sensitization" or "Sensitization, Immunological" or "Immunostimulation" or "Immunologic Stimulation" or "Sensitizations, Immunological" or "biologic response modifier therapy" or "biological response modifier therapy" or "brm therapy" or "immune therapy" or "immunisation" or "immunisation efficiency" or "immunisation prophylaxis" or "immunisation schedule" or "immunization" or "immunization efficiency" or "immunization prophylaxis" or "immunization schedule" or "immunogenic therapy" or "immunoglobulin therapy" or "immunologic sensitization" or "Immunological Stimulation" or "Immunological Stimulations" or "immunological therapy" or "immunological treatment" or "immunomodulant therapy" or "immunomodulary therapy" or "immunomodulating therapy" or "immunomodulation therapy" or "immunomodulative therapy" or "immunomodulator therapy" or "immunomodulatory intervention" or "immunomodulatory therapy" or "immunomoduling therapy" or "immunomodurating therapy" or "immunostimulation therapy" or "oral immunisation" or "oral immunization" or "reimmunisation" or "reimmunization" or "standard immunization program" or "Stimulation, Immunologic" or "Stimulation, Immunological" or "Stimulations, Immunological" or "Variolation" or "Variolations").ab,ti. |
| 8 | 4 or 5 or 6 or 7 |
| 9 | 3 and 8 |
| 10 | exp Haemophilus Vaccines/ |
| 11 | ("Non-typeable Haemophilus influenzae-Moraxella catarrhalis vaccine" or "Vaccines, Haemophilus" or "Hemophilus Vaccines" or "Vaccines, Hemophilus" or "Haemophilus influenzae Vaccines" or "Vaccines, Haemophilus influenzae" or "Haemophilus Vaccine" or "Non-typeable Haemophilus influenzae protein vaccine" or "inactivated nontypeable Haemophilus influenzae " or "non-typeable Haemophilus influenzae-Moraxella catarrhalis (NTHi-Mcat) vaccine" or "NTHi-Mcat vaccine" or "haemophilus vaccines" or "vaccine, haemophilus" or "hemophilus vaccine" or "hemophilus influenzae vaccine" or "hemophilus influenzae virus vaccine" or "Haemophilus influenzae vaccine").ab,ti. |
| 12 | 10 or 11 |
| 13 | 9 or 12 |
| 14 | exp Pulmonary Disease, Chronic Obstructive/ |
| 15 | ("Chronic Obstructive Lung Disease" or "Chronic Obstructive Pulmonary Diseases" or "COAD" or "COPD" or "Chronic Obstructive Airway Disease" or "Chronic Obstructive Pulmonary Disease" or "Airflow Obstruction, Chronic" or "Airflow Obstructions, Chronic" or "Chronic Airflow Obstructions" or "Chronic Airflow Obstruction" or "pulmonary disease, chronic obstructive" or "chronic airway obstruction" or "chronic obstructive bronchopulmonary disease" or "chronic obstructive lung disorder" or "chronic obstructive pulmonary disorder" or "chronic obstructive respiratory disease" or "chronic pulmonary obstructive disease" or "chronic pulmonary obstructive disorder" or "lung chronic obstructive disease" or "lung disease, chronic obstructive" or "obstructive chronic bronchitis" or "obstructive chronic lung disease" or "obstructive chronic pulmonary disease" or "obstructive lung disease, chronic" or "pulmonary disorder, chronic obstructive").ab,ti. |
| 16 | 14 or 15 |
| 17 | 13 and 16 |
| EBM Reviews - Cochrane Central Register of Controlled Trials <October 2022> | |
| 1 | exp haemophilus/ |
| 2 | ("Hemophilus" or "haemophilus" or "haemophilus isolation" or "Mycobacterium influenzae" or "Haemophilus meningitidis" or "Influenza-bacillus" or "Hemophilus influenzae" or "Coccobacillus pfeifferi" or "bacterium influenzae" or "h. influenzae" or "haemophilus influenza" or "hemophilus influenza extract" or "influenza bacillus" or "pfeiffer bacillus" or "haemophilus influenzae" or "non typeable haemophilus influenzae" or "Non-typeable Haemophilus influenza" or "NTHi-Mcat" or "nontypeable Haemophilus influenzae ").af. |
| 3 | 1 or 2 |
| 4 | exp immunization/ |
| 5 | exp immunotherapy/ |
| 6 | exp Vaccine/ |
| 7 | ("immunotherapy" or "Vaccines" or "Vaccine" or "Sensitization, Immunologic" or "Immunizations" or "Immunotherapies" or "Immunological Sensitizations" or "Immunological Sensitization" or "Sensitization, Immunological" or "Immunostimulation" or "Immunologic Stimulation" or "Sensitizations, Immunological" or "biologic response modifier therapy" or "biological response modifier therapy" or "brm therapy" or "immune therapy" or "immunisation" or "immunisation efficiency" or "immunisation prophylaxis" or "immunisation schedule" or "immunization" or "immunization efficiency" or "immunization prophylaxis" or "immunization schedule" or "immunogenic therapy" or "immunoglobulin therapy" or "immunologic sensitization" or "Immunological Stimulation" or "Immunological Stimulations" or "immunological therapy" or "immunological treatment" or "immunomodulant therapy" or "immunomodulary therapy" or "immunomodulating therapy" or "immunomodulation therapy" or "immunomodulative therapy" or "immunomodulator therapy" or "immunomodulatory intervention" or "immunomodulatory therapy" or "immunomoduling therapy" or "immunomodurating therapy" or "immunostimulation therapy" or "oral immunisation" or "oral immunization" or "reimmunisation" or "reimmunization" or "standard immunization program" or "Stimulation, Immunologic" or "Stimulation, Immunological" or "Stimulations, Immunological" or "Variolation" or "Variolations").ab,ti. |
| 8 | 4 or 5 or 6 or 7 |
| 9 | 3 and 8 |
| 10 | exp Haemophilus Vaccines/ |
| 11 | ("Non-typeable Haemophilus influenzae-Moraxella catarrhalis vaccine" or "Vaccines, Haemophilus" or "Hemophilus Vaccines" or "Vaccines, Hemophilus" or "Haemophilus influenzae Vaccines" or "Vaccines, Haemophilus influenzae" or "Haemophilus Vaccine" or "Non-typeable Haemophilus influenzae protein vaccine" or "inactivated nontypeable Haemophilus influenzae " or "non-typeable Haemophilus influenzae-Moraxella catarrhalis (NTHi-Mcat) vaccine" or "NTHi-Mcat vaccine" or "haemophilus vaccines" or "vaccine, haemophilus" or "hemophilus vaccine" or "hemophilus influenzae vaccine" or "hemophilus influenzae virus vaccine" or "Haemophilus influenzae vaccine").ab,ti. |
| 12 | 10 or 11 |
| 13 | 9 or 12 |
| 14 | exp Pulmonary Disease, Chronic Obstructive/ |
| 15 | ("Chronic Obstructive Lung Disease" or "Chronic Obstructive Pulmonary Diseases" or "COAD" or "COPD" or "Chronic Obstructive Airway Disease" or "Chronic Obstructive Pulmonary Disease" or "Airflow Obstruction, Chronic" or "Airflow Obstructions, Chronic" or "Chronic Airflow Obstructions" or "Chronic Airflow Obstruction" or "pulmonary disease, chronic obstructive" or "chronic airway obstruction" or "chronic obstructive bronchopulmonary disease" or "chronic obstructive lung disorder" or "chronic obstructive pulmonary disorder" or "chronic obstructive respiratory disease" or "chronic pulmonary obstructive disease" or "chronic pulmonary obstructive disorder" or "lung chronic obstructive disease" or "lung disease, chronic obstructive" or "obstructive chronic bronchitis" or "obstructive chronic lung disease" or "obstructive chronic pulmonary disease" or "obstructive lung disease, chronic" or "pulmonary disorder, chronic obstructive").ab,ti. |
| 16 | 14 or 15 |
| 17 | 13 and 16 |

| Pubmed |
| --- |
| ((((("Haemophilus"[Mesh]) OR ("Haemophilus influenzae"[Mesh])) OR ("Hemophilus"[Title/Abstract] OR "haemophilus"[Title/Abstract] OR "haemophilus isolation"[Title/Abstract] OR "Mycobacterium influenzae"[Title/Abstract] OR "Haemophilus meningitidis"[Title/Abstract] OR "Influenza-bacillus"[Title/Abstract] OR "Hemophilus influenzae"[Title/Abstract] OR "Coccobacillus pfeifferi"[Title/Abstract] OR "bacterium influenzae"[Title/Abstract] OR "h. influenzae"[Title/Abstract] OR "haemophilus influenza"[Title/Abstract] OR "hemophilus influenza extract"[Title/Abstract] OR "influenza bacillus"[Title/Abstract] OR "pfeiffer bacillus"[Title/Abstract] OR "haemophilus influenzae"[Title/Abstract] OR "non typeable haemophilus influenzae"[Title/Abstract] OR "Non-typeable Haemophilus influenza"[Title/Abstract] OR "NTHi-Mcat"[Title/Abstract] OR "nontypeable Haemophilus influenzae "[Title/Abstract])) AND ("immunotherapy"[Title/Abstract] OR "Vaccines"[Title/Abstract] OR "Vaccine"[Title/Abstract] OR "Sensitization, Immunologic"[Title/Abstract] OR "Immunizations"[Title/Abstract] OR "Immunotherapies"[Title/Abstract] OR "Immunological Sensitizations"[Title/Abstract] OR "Immunological Sensitization"[Title/Abstract] OR "Sensitization, Immunological"[Title/Abstract] OR "Immunostimulation"[Title/Abstract] OR "Immunologic Stimulation"[Title/Abstract] OR "Sensitizations, Immunological"[Title/Abstract] OR "biologic response modifier therapy"[Title/Abstract] OR "biological response modifier therapy"[Title/Abstract] OR "brm therapy"[Title/Abstract] OR "immune therapy"[Title/Abstract] OR "immunisation"[Title/Abstract] OR "immunisation efficiency"[Title/Abstract] OR "immunisation prophylaxis"[Title/Abstract] OR "immunisation schedule"[Title/Abstract] OR "immunization"[Title/Abstract] OR "immunization efficiency"[Title/Abstract] OR "immunization prophylaxis"[Title/Abstract] OR "immunization schedule"[Title/Abstract] OR "immunogenic therapy"[Title/Abstract] OR "immunoglobulin therapy"[Title/Abstract] OR "immunologic sensitization"[Title/Abstract] OR "Immunological Stimulation"[Title/Abstract] OR "Immunological Stimulations"[Title/Abstract] OR "immunological therapy"[Title/Abstract] OR "immunological treatment"[Title/Abstract] OR "immunomodulant therapy"[Title/Abstract] OR "immunomodulary therapy"[Title/Abstract] OR "immunomodulating therapy"[Title/Abstract] OR "immunomodulation therapy"[Title/Abstract] OR "immunomodulative therapy"[Title/Abstract] OR "immunomodulator therapy"[Title/Abstract] OR "immunomodulatory intervention"[Title/Abstract] OR "immunomodulatory therapy"[Title/Abstract] OR "immunomoduling therapy"[Title/Abstract] OR "immunomodurating therapy"[Title/Abstract] OR "immunostimulation therapy"[Title/Abstract] OR "oral immunisation"[Title/Abstract] OR "oral immunization"[Title/Abstract] OR "reimmunisation"[Title/Abstract] OR "reimmunization"[Title/Abstract] OR "standard immunization program"[Title/Abstract] OR "Stimulation, Immunologic"[Title/Abstract] OR "Stimulation, Immunological"[Title/Abstract] OR "Stimulations, Immunological"[Title/Abstract] OR "Variolation"[Title/Abstract] OR "Variolations"[Title/Abstract])) OR (("Haemophilus Vaccines"[Mesh]) OR ("Non-typeable Haemophilus influenzae-Moraxella catarrhalis vaccine"[Title/Abstract] OR "Vaccines, Haemophilus"[Title/Abstract] OR "Hemophilus Vaccines"[Title/Abstract] OR "Vaccines, Hemophilus"[Title/Abstract] OR "Haemophilus influenzae Vaccines"[Title/Abstract] OR "Vaccines, Haemophilus influenzae"[Title/Abstract] OR "Haemophilus Vaccine"[Title/Abstract] OR "Non-typeable Haemophilus influenzae protein vaccine"[Title/Abstract] OR "inactivated nontypeable Haemophilus influenzae "[Title/Abstract] OR "non-typeable Haemophilus influenzae-Moraxella catarrhalis (NTHi-Mcat) vaccine"[Title/Abstract] OR "NTHi-Mcat vaccine"[Title/Abstract] OR "haemophilus vaccines"[Title/Abstract] OR "vaccine, haemophilus"[Title/Abstract] OR "hemophilus vaccine"[Title/Abstract] OR "hemophilus influenzae vaccine"[Title/Abstract] OR "hemophilus influenzae virus vaccine"[Title/Abstract] OR "Haemophilus influenzae vaccine"[Title/Abstract]))) AND (("Pulmonary Disease, Chronic Obstructive"[Mesh]) OR ("Chronic Obstructive Lung Disease"[Title/Abstract] OR "Chronic Obstructive Pulmonary Diseases"[Title/Abstract] OR "COAD"[Title/Abstract] OR "COPD"[Title/Abstract] OR "Chronic Obstructive Airway Disease"[Title/Abstract] OR "Chronic Obstructive Pulmonary Disease"[Title/Abstract] OR "Airflow Obstruction, Chronic"[Title/Abstract] OR "Airflow Obstructions, Chronic"[Title/Abstract] OR "Chronic Airflow Obstructions"[Title/Abstract] OR "Chronic Airflow Obstruction"[Title/Abstract] OR "pulmonary disease, chronic obstructive"[Title/Abstract] OR "chronic airway obstruction"[Title/Abstract] OR "chronic obstructive bronchopulmonary disease"[Title/Abstract] OR "chronic obstructive lung disorder"[Title/Abstract] OR "chronic obstructive pulmonary disorder"[Title/Abstract] OR "chronic obstructive respiratory disease"[Title/Abstract] OR "chronic pulmonary obstructive disease"[Title/Abstract] OR "chronic pulmonary obstructive disorder"[Title/Abstract] OR "lung chronic obstructive disease"[Title/Abstract] OR "lung disease, chronic obstructive"[Title/Abstract] OR "obstructive chronic bronchitis"[Title/Abstract] OR "obstructive chronic lung disease"[Title/Abstract] OR "obstructive chronic pulmonary disease"[Title/Abstract] OR "obstructive lung disease, chronic"[Title/Abstract] OR "pulmonary disorder, chronic obstructive"[Title/Abstract])) |

## Table S2. List of studies excluded via full-text assessment and studies included in the analyses.

| 1. Non-RCT (n=3) ^1-3^ |
| --- |
| 2. No available data (n=6) ^4-9^ |
| 3. Duplicated population (n=6) ^10-15^ |
| 4. Full text unavailable(n=3) ^16-18^ |
| 5. Repeat(n=1) ^19^ |

1. Clancy RL, Dunkley M. Acute exacerbations in COPD and their control with oral immunization with non-typeable haemophilus influenzae. Frontiers in immunology 2011;2:7. doi: 10.3389/fimmu.2011.00007.

2. Clancy RL, Dunkley M. A vaccine to prevent exacerbations in COPD. The Medical journal of Australia 2011;195:99-100. doi: 10.5694/j.1326-5377.2011.tb03224.x.

3. Brown HM, Wilson RN. Chronic bronchitis in industry; an account of a trial of H. influenzae vaccine. British medical journal 1959;1:263-7. doi: 10.1136/bmj.1.5117.263.

4. An oral killed non-typeable Haemophilus influenzae vaccine for preventing episodes of acute bronchitis in patients with mild to moderate airways disease: safety and efficacy study. 2006

5. A Multi-Centre, Randomised, Double-Blind, Placebo-Controlled, Parallel Group, Single-Season Study to Assess the Efficacy and Safety of HI-164OV Oral Vaccine in Moderate to Severe chronic obstructive pulmonary disease (COPD). 2010

6. Study to Assess the Safety of a New GSK Biologicals' GSK2231395A Candidate Vaccine. 2009

7. Clancy RL, Dunkley ML. Oral non-typable Haemophilus influenzae enhances physiological mechanism of airways protection. Clinical and experimental immunology 2010;161:127-33. doi: 10.1111/j.1365-2249.2010.04142.x.

8. Study to assess the immunogenicity and safety of GSK's investigational vaccine (GSK3277511A) when given to healthy smokers and ex-smokers after Shingrix vaccination. 2019

9. Galgani I, Annaratone M, Casula D, et al. Safety and immunogenicity of three doses of non-typeable Haemophilus influenzae-Moraxella catarrhalis (NTHi-Mcat) vaccine when administered according to two different schedules: a phase 2, randomised, observer-blind study. Respiratory research 2022;23:114. doi: 10.1186/s12931-022-02019-4.

10. A Study to Evaluate the Safety, Reactogenicity and Immunogenicity of GlaxoSmithkline (GSK) Biologicals' Investigational Vaccine GSK2838504A When Administered to Chronic Obstructive Pulmonary Disease (COPD) Patients With Persistent Airflow Obstruction. 2014

11. Wilkinson T, Schembri S, Brightling C, et al. Late breaking abstract-safety and immunogenicity of non-typeable H. influenzae (NTHi) adjuvanted vaccine in older adults with chronic obstructive pulmonary disease (COPD). European respiratory journal 2018;52:2018-09. doi: <https://doi.org/10.1183/13993003.congress-2018.PA4089>.

12. Van Damme P, Leroux-Roels G, Vandermeulen C, et al. Late breaking abstract-safety and immunogenicity of non-typeable H. influenzae (NTHi) and M. catarrhalis (MCAT) adjuvanted vaccines in adults. European respiratory journal 2018;52 doi: <https://doi.org/10.1183/13993003.congress-2018.PA4088>.

13. Riccucci D, Wilkinson T, Schembri S, et al. Clinical impact of a non-typeable Haemophilus influenzae (NTHi) adjuvanted vaccine in adults with chronic obstructive pulmonary disease (COPD). European respiratory journal 2019;54:2019-09. doi: <https://doi.org/10.1183/13993003.congress-2019.OA264>.

14. Andreas S, Testa M, Boyer L, et al. First-time assessment of efficacy of candidate vaccine to prevent acute exacerbations of chronic obstructive pulmonary disease (AECOPD): multicentre, randomised, controlled, observer-blind phase 2b trial. European Respiratory Journal Conference: International Congress of the European Respiratory Society, ERS 2021;58 doi: <https://dx.doi.org/10.1183/13993003.congress-2021.RCT210>.

15. Arora AK, Chinsky K, Keller C, et al. A detailed analysis of possible efficacy signals of NTHi-Mcat vaccine against severe COPD exacerbations in a previously reported randomised phase 2b trial. Vaccine 2022;40:5924-32. doi: 10.1016/j.vaccine.2022.08.053.

16. Halasa J, Halasa M, Wojciechowska W, et al. Clinical efficacy of autovaccine in the treatment of infectious nonatopic asthma and COPD - Double blind placebo controlled trial. [Polish]. Alergia Astma Immunologia 2001;6(2):109-13.

17. Tandon MK. Double-blind controlled trial of oral immunization with killed H Influenzae (Bronchostat) in elderly COAD patients. Aust NZ j med suppl 1989;Vol.19:650p.

18. Antonova LP, Romanov VV, Averbakh MM. [Experience with bronchomunal used in the combined treatment of patients with bronchial asthma and chronic obstructive pulmonary disease]. Problemy tuberkuleza i boleznei legkikh 2008:8-11.

19. A study to test if the vaccine is working well in Chronic Obstructive Pulmonary Disease (COPD) patients aged 40 to 80 years old to reduce episodes of worsening symptoms and to gather further information on safety and immune response. 2017

## Table S3. The characteristics of included studies.

| **First author** | **Year** | **Intervention** | | |  | **Control** | |
| --- | --- | --- | --- | --- | --- | --- | --- |
|  |  | **Containing of Interventions** | **Duration (days)** | **Dose of medication** |  | **Duration (days)** | **Dose of medication** |
| Andreas S. | 2022 | The NTHi–Mcat vaccine contained 10 µg protein D, 10 µg PE–PilA, and 3·3 µg UspA2, and included Adjuvant System AS01E, containing 3-O-desacyl-4ʹ-monophosphoryl lipid A (MPL), QS-21 (Quillaja saponaria Molina, fraction 21; licensed by GSK from Antigenics LLC, a wholly owned subsidiary of Agenus Inc, a Delaware USA corporation) and liposome (25 µg MPL and 25 µg QS-21). | 0-60 | NR |  | 0-60 | NR |
| Clancy R. | 1985 | Enteric-coated tablets each containing 10^11^ killed H influenzae with sodium tauroglycocholate | 0-28-56 | Each course consisted of two tablets taken before breakfast each day for 3 consecutive days |  | 0-28-56 | Each course was 2 tablets taken before breakfast on 3 consecutive days |
|  |  | Enteric-coated tablets each containing 10^11^ killed H influenzae with sodium tauroglycocholate | 0-28-56 | Each course consisted of two tablets taken before breakfast each day for 3 consecutive days |  | 0-28-56 | Each course was 2 tablets taken before breakfast on 3 consecutive days |
| Clancy R. L. | 2016 | Approximately 2×10^11^ NTHi-164 of formalin-inactivated NTHi (HI-164) | 0-28-56 | 45 mg (Each course was two tablets daily (before breakfast) for 3 consecutive days) |  | 0-28-56 | Each course was two tablets daily (before breakfast) for 3 consecutive days |
| De Smedt P. | 2021 | Containing 10 mg PD, 10 mg E-PilA and 10 mg UspA2 (10–10-AS01) | 0-60 | NR |  | 0-60 | NR |
|  |  | Containing 10 mg PD, 10 mg PE-PilA and 3.3 mg UspA2 (10–3-AS01) | 0-61 | NR |  | 0-60 | NR |
| Philips M. | 2007 | HI-1-164-AS | NR | NR |  | NR | NR |
| Tandon M. K. | 2010 | Containing 45 mg (approximately 10^11^ bacteria) of formalin-inactivated NTHi | 0-28-56 | 45 mg (each course consisted of two tablets daily for three con_x005fsecutive days (befo re breakfast)) |  | 0-28-56 | 45 mg (Each course consisted of two tablets daily for three con_x005fsecutive days (befo re breakfast) |
| Van Damme P. | 2019 | Containing 10 mg PD, 10 mg PE-PilA (fusion protein)and 10 mg UspA2, with adjuvantation (AS01E) | 0-60 | NR |  | 0-60 | NR |
|  |  | Containing 10 mg PD, 10 mg PE-PilA (fusion protein) and 3.3 mg UspA2, with adjuvantation (AS01E). | 0-60 | NR |  | 0-60 | NR |
| Wilkinson T. M. A. | 2019 | Containing 10 mg PD and 10 mg PE-PilA per dose with AS01E (containing immunostimulants, 3-O-desacyl-40-monophosphoryl lipid A and saponin QS-21, 25 mg each, and liposomes), as described previously | 0-60 | NR |  | 0-60 | NR |

## Table S4. Summary of safety of NTHi vaccine in COPD patients.

| Author | Year | System | Adverse Event | Intervention | | Control | |
| --- | --- | --- | --- | --- | --- | --- | --- |
|  |  |  |  | No. events | N | No. events | N |
| Andreas S._2 | 2022 | Blood and lymphatic system disorders | Anaemia | 0 | 304 | 302 | 1 |
| Van Damme P._1 | 2019 | Blood and lymphatic system disorders-Infections and infestations | Lymph gland infection | 0 | 30 | 44 | 0 |
| Van Damme P._2 | 2019 | Blood and lymphatic system disorders-Infections and infestations | Lymph gland infection | 0 | 31 | 44 | 0 |
| Andreas S._2 | 2022 | Blood and lymphatic system disorders-Infections and infestations | Sepsis | 0 | 304 | 302 | 1 |
| Andreas S._2 | 2022 | Blood and lymphatic system disorders-Infections and infestations | Septic shock | 1 | 304 | 302 | 0 |
| Andreas S._2 | 2022 | Cardiac disorders | Acute left ventricular failure | 0 | 304 | 302 | 1 |
| Andreas S._2 | 2022 | Cardiac disorders | Acute myocardial infarction | 2 | 304 | 302 | 1 |
| Andreas S._2 | 2022 | Cardiac disorders | Angina pectoris | 1 | 304 | 302 | 1 |
| Andreas S._2 | 2022 | Cardiac disorders | Angina unstable | 0 | 304 | 302 | 1 |
| Andreas S._2 | 2022 | Cardiac disorders | Arrhythmia | 0 | 304 | 302 | 1 |
| Andreas S._2 | 2022 | Cardiac disorders | Atrial fibrillation | 4 | 304 | 302 | 2 |
| Andreas S._2 | 2022 | Cardiac disorders | Atrial fibrillation | 2 | 304 | 302 | 0 |
| Andreas S._2 | 2022 | Cardiac disorders | Atrial flutter | 1 | 304 | 302 | 0 |
| Andreas S._2 | 2022 | Cardiac disorders | Cardiac failure | 0 | 304 | 302 | 2 |
| Andreas S._2 | 2022 | Cardiac disorders | Cardiac failure | 0 | 304 | 302 | 1 |
| Andreas S._2 | 2022 | Cardiac disorders | Cardiac failure congestive | 1 | 304 | 302 | 2 |
| Andreas S._2 | 2022 | Cardiac disorders | Cardiomyopathy | 0 | 304 | 302 | 1 |
| Andreas S._2 | 2022 | Cardiac disorders | Coronary artery insufficiency | 0 | 304 | 302 | 1 |
| Andreas S._2 | 2022 | Cardiac disorders | Mitral valve prolapse | 0 | 304 | 302 | 1 |
| Andreas S._2 | 2022 | Cardiac disorders | Pericardial effusion | 0 | 304 | 302 | 1 |
| Andreas S._2 | 2022 | Cardiac disorders | Sinus node dysfunction | 0 | 304 | 302 | 1 |
| Andreas S._2 | 2022 | Cardiac disorders | Stress cardiomyopathy | 1 | 304 | 302 | 1 |
| Andreas S._2 | 2022 | Cardiac disorders | Supraventricular tachycardia | 1 | 304 | 302 | 0 |
| Andreas S._2 | 2022 | Cardiac disorders | Trifascicular block | 1 | 304 | 302 | 0 |
| Andreas S._2 | 2022 | Cardiac disorders | Ventricular tachycardia | 0 | 304 | 302 | 1 |
| Wilkinson T. M. A. | 2019 | Cardiac disorders | Atrial fibrillation | 1 | 73 | 72 | 0 |
| Wilkinson T. M. A. | 2019 | Cardiac disorders | Bradycardia | 0 | 73 | 72 | 1 |
| Wilkinson T. M. A. | 2019 | Cardiac disorders | Cardiac failure | 0 | 73 | 72 | 1 |
| Wilkinson T. M. A. | 2019 | Cardiac disorders | Dressler's syndrome | 0 | 73 | 72 | 1 |
| Wilkinson T. M. A. | 2019 | Cardiac disorders | Myocardial infarction | 1 | 73 | 72 | 2 |
| Andreas S._2 | 2022 | Ear and labyrinth disorders | Cataract | 1 | 304 | 302 | 0 |
| Andreas S._2 | 2022 | Ear and labyrinth disorders | Cerumen impaction | 1 | 304 | 302 | 0 |
| Andreas S._2 | 2022 | Ear and labyrinth disorders | Dermatochalasis | 1 | 304 | 302 | 0 |
| Andreas S._2 | 2022 | Ear and labyrinth disorders | Ear pain | 1 | 304 | 302 | 1 |
| Andreas S._2 | 2022 | Ear and labyrinth disorders | Ocular hyperaemia | 0 | 304 | 302 | 1 |
| Andreas S._2 | 2022 | Ear and labyrinth disorders | Retinoschisis | 1 | 304 | 302 | 0 |
| Andreas S._2 | 2022 | Ear and labyrinth disorders | Vertigo | 0 | 304 | 302 | 1 |
| Andreas S._2 | 2022 | Ear and labyrinth disorders | Vertigo | 0 | 304 | 302 | 2 |
| Andreas S._2 | 2022 | Ear and labyrinth disorders | Vision blurred | 0 | 304 | 302 | 1 |
| Andreas S._2 | 2022 | Ear and labyrinth disorders | Vitreous detachment | 1 | 304 | 302 | 0 |
| Andreas S._2 | 2022 | Ear and labyrinth disorders-Infections and infestations | Conjunctivitis | 0 | 304 | 302 | 1 |
| Andreas S._2 | 2022 | Ear and labyrinth disorders-Infections and infestations | Ear infection | 0 | 304 | 302 | 1 |
| Andreas S._2 | 2022 | Ear and labyrinth disorders-Infections and infestations | Otitis media acute | 0 | 304 | 302 | 1 |
| Andreas S._2 | 2022 | Gastrointestinal disorders | Abdominal discomfort | 1 | 304 | 302 | 0 |
| Andreas S._2 | 2022 | Gastrointestinal disorders | Abdominal pain | 1 | 304 | 302 | 0 |
| Andreas S._2 | 2022 | Gastrointestinal disorders | Abdominal pain lower | 0 | 304 | 302 | 1 |
| Van Damme P._1 | 2019 | Gastrointestinal disorders | Abdominal pain upper | 0 | 30 | 44 | 0 |
| Van Damme P._2 | 2019 | Gastrointestinal disorders | Abdominal pain upper | 0 | 31 | 44 | 0 |
| Andreas S._2 | 2022 | Gastrointestinal disorders | Abdominal pain upper | 0 | 304 | 302 | 1 |
| Wilkinson T. M. A. | 2019 | Gastrointestinal disorders | Barrett's oesophagus | 0 | 73 | 72 | 1 |
| Wilkinson T. M. A. | 2019 | Gastrointestinal disorders | Constipation | 1 | 73 | 72 | 0 |
| Andreas S._2 | 2022 | Gastrointestinal disorders | Constipation | 0 | 304 | 302 | 1 |
| Andreas S._2 | 2022 | Gastrointestinal disorders | Dental caries | 2 | 304 | 302 | 0 |
| Andreas S._2 | 2022 | Gastrointestinal disorders | Diarrhoea | 3 | 304 | 302 | 9 |
| Van Damme P._2 | 2019 | Gastrointestinal disorders | Diarrhoea | 1 | 31 | 44 | 1 |
| Van Damme P._1 | 2019 | Gastrointestinal disorders | Diarrhoea | 0 | 30 | 44 | 1 |
| Andreas S._2 | 2022 | Gastrointestinal disorders | Dyschezia | 0 | 304 | 302 | 1 |
| Andreas S._2 | 2022 | Gastrointestinal disorders | Enterovesical fistula | 0 | 304 | 302 | 1 |
| Andreas S._2 | 2022 | Gastrointestinal disorders | Faecaloma | 0 | 304 | 302 | 1 |
| Van Damme P._1 | 2019 | Gastrointestinal disorders | Gastrointestinal pain | 0 | 30 | 44 | 0 |
| Van Damme P._2 | 2019 | Gastrointestinal disorders | Gastrointestinal pain | 0 | 31 | 44 | 0 |
| Andreas S._2 | 2022 | Gastrointestinal disorders | Gastrooesophageal reflux disease | 1 | 304 | 302 | 1 |
| De Smedt P._2 | 2021 | Gastrointestinal disorders | Ileus | 1 | 27 | 28 | 0 |
| Andreas S._2 | 2022 | Gastrointestinal disorders | Ileus | 0 | 304 | 302 | 1 |
| De Smedt P._1 | 2021 | Gastrointestinal disorders | Ileus | 0 | 26 | 28 | 0 |
| Andreas S._2 | 2022 | Gastrointestinal disorders | Ileus paralytic | 1 | 304 | 302 | 0 |
| De Smedt P._2 | 2021 | Gastrointestinal disorders | Ileus paralytic | 1 | 27 | 28 | 0 |
| De Smedt P._1 | 2021 | Gastrointestinal disorders | Ileus paralytic | 0 | 26 | 28 | 0 |
| Andreas S._2 | 2022 | Gastrointestinal disorders | Inguinal hernia | 0 | 304 | 302 | 1 |
| Van Damme P._1 | 2019 | Gastrointestinal disorders | Inguinal hernia | 0 | 30 | 44 | 1 |
| Van Damme P._2 | 2019 | Gastrointestinal disorders | Inguinal hernia | 0 | 31 | 44 | 1 |
| De Smedt P._2 | 2021 | Gastrointestinal disorders | Intestinal obstruction | 1 | 27 | 28 | 0 |
| De Smedt P._1 | 2021 | Gastrointestinal disorders | Intestinal obstruction | 0 | 26 | 28 | 0 |
| Andreas S._2 | 2022 | Gastrointestinal disorders | Intestinal polyp | 1 | 304 | 302 | 0 |
| Andreas S._2 | 2022 | Gastrointestinal disorders | Intestinal pseudo-obstruction | 1 | 304 | 302 | 0 |
| Andreas S._2 | 2022 | Gastrointestinal disorders | Lower gastrointestinal haemorrhage | 0 | 304 | 302 | 1 |
| Andreas S._2 | 2022 | Gastrointestinal disorders | Nausea | 1 | 304 | 302 | 0 |
| Andreas S._2 | 2022 | Gastrointestinal disorders | Nausea | 1 | 304 | 302 | 3 |
| Andreas S._2 | 2022 | Gastrointestinal disorders | Pancreatic disorder | 1 | 304 | 302 | 0 |
| Andreas S._2 | 2022 | Gastrointestinal disorders | Retroperitoneal haematoma | 1 | 304 | 302 | 0 |
| Andreas S._2 | 2022 | Gastrointestinal disorders | Toothache | 2 | 304 | 302 | 0 |
| Andreas S._2 | 2022 | Gastrointestinal disorders | Vomiting | 1 | 304 | 302 | 1 |
| Andreas S._2 | 2022 | Gastrointestinal disorders | Vomiting | 1 | 304 | 302 | 1 |
| Andreas S._2 | 2022 | Gastrointestinal disorders-Infections and infestations | Abdominal abscess | 0 | 304 | 302 | 1 |
| Andreas S._2 | 2022 | Gastrointestinal disorders-Infections and infestations | Enterococcal infection | 1 | 304 | 302 | 0 |
| Andreas S._2 | 2022 | Gastrointestinal disorders-Infections and infestations | Enterocolitis viral | 1 | 304 | 302 | 0 |
| Andreas S._2 | 2022 | Gastrointestinal disorders-injury, poisoning and procedural complications | Abdominal wound dehiscence | 0 | 304 | 302 | 1 |
| Andreas S._2 | 2022 | General disorders | Asthenia | 0 | 304 | 302 | 1 |
| Andreas S._1 | 2022 | General disorders | Chills | 3 | 301 | 299 | 1 |
| Andreas S._2 | 2022 | General disorders | Condition aggravated | 0 | 304 | 302 | 1 |
| Andreas S._2 | 2022 | General disorders | Drug intolerance | 0 | 304 | 302 | 1 |
| Andreas S._2 | 2022 | General disorders | Feeling hot | 0 | 304 | 302 | 1 |
| Andreas S._2 | 2022 | General disorders | Injection site bruising | 0 | 304 | 302 | 1 |
| Andreas S._2 | 2022 | General disorders | Injection site reaction | 1 | 304 | 302 | 0 |
| Andreas S._2 | 2022 | General disorders | Malaise | 0 | 304 | 302 | 1 |
| Van Damme P._1 | 2019 | General disorders | Malaise | 0 | 30 | 44 | 0 |
| Van Damme P._2 | 2019 | General disorders | Malaise | 0 | 31 | 44 | 0 |
| Andreas S._2 | 2022 | General disorders | Oedema | 1 | 304 | 302 | 0 |
| Andreas S._2 | 2022 | General disorders | Oedema peripheral | 5 | 304 | 302 | 0 |
| Andreas S._2 | 2022 | General disorders | Oxygen saturation decreased | 0 | 304 | 302 | 1 |
| Van Damme P._1 | 2019 | General disorders | Temperature (Oral) (°C) | 3 | 29 | 44 | 1 |
| Van Damme P._2 | 2019 | General disorders | Temperature (Oral) (°C) | 3 | 31 | 44 | 1 |
| Van Damme P._1 | 2019 | General disorders | Temperature (Oral) (°C) | 2 | 30 | 44 | 0 |
| Van Damme P._2 | 2019 | General disorders | Temperature (Oral) (°C) | 0 | 31 | 44 | 0 |
| Wilkinson T. M. A. | 2019 | General disorders | Tooth abscess | 1 | 73 | 72 | 0 |
| Andreas S._2 | 2022 | General disorders | Weight decreased | 0 | 304 | 302 | 1 |
| Andreas S._2 | 2022 | General disorders | Weight increased | 0 | 304 | 302 | 1 |
| Andreas S._2 | 2022 | Hepatobiliary disorders | Cholecystitis acute | 1 | 304 | 302 | 0 |
| Wilkinson T. M. A. | 2019 | Hepatobiliary disorders | Cholecystitis | 1 | 73 | 72 | 0 |
| Andreas S._2 | 2022 | Hepatobiliary disorders | Cholelithiasis | 0 | 304 | 302 | 1 |
| Andreas S._2 | 2022 | Hepatobiliary disorders | Cirrhosis alcoholic | 1 | 304 | 302 | 0 |
| Andreas S._2 | 2022 | Immune system disorders | Seasonal allergy | 1 | 304 | 302 | 1 |
| Wilkinson T. M. A. | 2019 | Infections and infestations | Cellulitis | 1 | 73 | 72 | 0 |
| Andreas S._2 | 2022 | Infections and infestations | Diverticulitis | 0 | 304 | 302 | 1 |
| Andreas S._2 | 2022 | Infections and infestations | Erysipelas | 0 | 304 | 302 | 1 |
| Andreas S._2 | 2022 | Infections and infestations | Fungal infection | 0 | 304 | 302 | 1 |
| Andreas S._2 | 2022 | Infections and infestations | Graft infection | 1 | 304 | 302 | 0 |
| Andreas S._2 | 2022 | Infections and infestations | Herpes zoster | 0 | 304 | 302 | 1 |
| Andreas S._2 | 2022 | Infections and infestations | Infection | 1 | 304 | 302 | 0 |
| Andreas S._2 | 2022 | Infections and infestations | Infective exacerbation of chronic obstructive airways disease | 1 | 304 | 302 | 4 |
| Wilkinson T. M. A. | 2019 | Infections and infestations | Infective exacerbation of chronic obstructive airways disease | 2 | 73 | 72 | 2 |
| Andreas S._2 | 2022 | Infections and infestations | Influenza | 4 | 304 | 302 | 2 |
| Andreas S._2 | 2022 | Infections and infestations | Influenza | 2 | 304 | 302 | 3 |
| Van Damme P._2 | 2019 | Infections and infestations | Influenza | 2 | 31 | 44 | 2 |
| Van Damme P._1 | 2019 | Infections and infestations | Influenza | 0 | 30 | 44 | 2 |
| Andreas S._2 | 2022 | Infections and infestations | Labyrinthitis | 1 | 304 | 302 | 0 |
| Andreas S._2 | 2022 | Infections and infestations | Laryngitis | 0 | 304 | 302 | 1 |
| Andreas S._2 | 2022 | Infections and infestations | Localised infection | 1 | 304 | 302 | 0 |
| Andreas S._2 | 2022 | Infections and infestations | Paronychia | 1 | 304 | 302 | 0 |
| Andreas S._2 | 2022 | Infections and infestations | Pharyngitis | 1 | 304 | 302 | 0 |
| Van Damme P._1 | 2019 | Infections and infestations | Rhinitis | 2 | 30 | 44 | 1 |
| Andreas S._2 | 2022 | Infections and infestations | Rhinitis | 0 | 304 | 302 | 1 |
| Van Damme P._2 | 2019 | Infections and infestations | Rhinitis | 0 | 31 | 44 | 1 |
| Andreas S._2 | 2022 | Infections and infestations | Sweat gland infection | 1 | 304 | 302 | 0 |
| Andreas S._2 | 2022 | Infections and infestations | Viral infection | 0 | 304 | 302 | 1 |
| Andreas S._2 | 2022 | Injury, poisoning and procedural complications | Alcohol poisoning | 1 | 304 | 302 | 0 |
| Andreas S._2 | 2022 | Injury, poisoning and procedural complications | Anastomotic complication | 0 | 304 | 302 | 1 |
| Van Damme P._1 | 2019 | Injury, poisoning and procedural complications | Ankle fracture | 0 | 30 | 44 | 1 |
| Van Damme P._2 | 2019 | Injury, poisoning and procedural complications | Ankle fracture | 0 | 31 | 44 | 1 |
| Andreas S._2 | 2022 | Injury, poisoning and procedural complications | Arthropod bite | 0 | 304 | 302 | 1 |
| Andreas S._2 | 2022 | Injury, poisoning and procedural complications | Contusion | 2 | 304 | 302 | 0 |
| Andreas S._2 | 2022 | Injury, poisoning and procedural complications | Drain site complication | 0 | 304 | 302 | 1 |
| Andreas S._2 | 2022 | Injury, poisoning and procedural complications | Exposure to toxic agent | 1 | 304 | 302 | 0 |
| Andreas S._2 | 2022 | Injury, poisoning and procedural complications | Fall | 1 | 304 | 302 | 0 |
| Andreas S._2 | 2022 | Injury, poisoning and procedural complications | Fall | 0 | 304 | 302 | 1 |
| Andreas S._2 | 2022 | Injury, poisoning and procedural complications | Femoral neck fracture | 0 | 304 | 302 | 1 |
| Andreas S._2 | 2022 | Injury, poisoning and procedural complications | Femur fracture | 0 | 304 | 302 | 1 |
| Andreas S._2 | 2022 | Injury, poisoning and procedural complications | Head injury | 0 | 304 | 302 | 1 |
| Andreas S._2 | 2022 | Injury, poisoning and procedural complications | Hip fracture | 0 | 304 | 302 | 1 |
| Andreas S._2 | 2022 | Injury, poisoning and procedural complications | Intentional overdose | 0 | 304 | 302 | 1 |
| Andreas S._2 | 2022 | Injury, poisoning and procedural complications | Joint injury | 1 | 304 | 302 | 0 |
| Andreas S._2 | 2022 | Injury, poisoning and procedural complications | Ligament rupture | 0 | 304 | 302 | 1 |
| Van Damme P._1 | 2019 | Injury, poisoning and procedural complications | Limb injury | 0 | 30 | 44 | 0 |
| Van Damme P._2 | 2019 | Injury, poisoning and procedural complications | Limb injury | 0 | 31 | 44 | 0 |
| Andreas S._2 | 2022 | Injury, poisoning and procedural complications | Meniscus injury | 1 | 304 | 302 | 0 |
| Andreas S._2 | 2022 | Injury, poisoning and procedural complications | Musculoskeletal procedural complication | 1 | 304 | 302 | 0 |
| Andreas S._2 | 2022 | Injury, poisoning and procedural complications | Post procedural haematoma | 0 | 304 | 302 | 1 |
| Andreas S._2 | 2022 | Injury, poisoning and procedural complications | Procedural pneumothorax | 0 | 304 | 302 | 1 |
| Andreas S._2 | 2022 | Injury, poisoning and procedural complications | Radius fracture | 0 | 304 | 302 | 1 |
| Wilkinson T. M. A. | 2019 | Injury, poisoning and procedural complications | Radius fracture | 0 | 73 | 72 | 1 |
| Andreas S._2 | 2022 | Injury, poisoning and procedural complications | Skin laceration | 0 | 304 | 302 | 1 |
| Andreas S._2 | 2022 | Injury, poisoning and procedural complications | Thermal burn | 1 | 304 | 302 | 0 |
| Andreas S._2 | 2022 | Injury, poisoning and procedural complications | Thoracic vertebral fracture | 1 | 304 | 302 | 0 |
| Andreas S._2 | 2022 | Injury, poisoning and procedural complications | Toxicity to various agents | 1 | 304 | 302 | 0 |
| De Smedt P._2 | 2021 | Injury, poisoning and procedural complications | Humerus fracture | 1 | 27 | 28 | 0 |
| De Smedt P._1 | 2021 | Injury, poisoning and procedural complications | Humerus fracture | 0 | 26 | 28 | 0 |
| De Smedt P._2 | 2021 | Injury, poisoning and procedural complications | Post-procedural fever | 1 | 27 | 28 | 0 |
| De Smedt P._1 | 2021 | Injury, poisoning and procedural complications | Post-procedural fever | 0 | 26 | 28 | 0 |
| De Smedt P._2 | 2021 | Injury, poisoning and procedural complications | Tendon rupture | 1 | 27 | 28 | 0 |
| De Smedt P._1 | 2021 | Injury, poisoning and procedural complications | Tendon rupture | 0 | 26 | 28 | 0 |
| De Smedt P._1 | 2021 | Injury, poisoning and procedural complications | Tibia fracture | 0 | 26 | 28 | 1 |
| De Smedt P._2 | 2021 | Injury, poisoning and procedural complications | Tibia fracture | 0 | 27 | 28 | 1 |
| De Smedt P._2 | 2021 | Injury, poisoning and procedural complications-Infections and infestations | Post-operative wound infection | 1 | 27 | 28 | 0 |
| De Smedt P._1 | 2021 | Injury, poisoning and procedural complications-Infections and infestations | Post-operative wound infection | 0 | 26 | 28 | 0 |
| Andreas S._2 | 2022 | Injury, poisoning and procedural complications-Infections and infestations | Wound abscess | 0 | 304 | 302 | 1 |
| Andreas S._2 | 2022 | Metabolism and nutrition disorders | Acidosis | 1 | 304 | 302 | 0 |
| Andreas S._2 | 2022 | Metabolism and nutrition disorders | Diabetic metabolic decompensation | 1 | 304 | 302 | 0 |
| Andreas S._2 | 2022 | Metabolism and nutrition disorders | Goitre | 0 | 304 | 302 | 1 |
| Andreas S._2 | 2022 | Metabolism and nutrition disorders | Gout | 1 | 304 | 302 | 0 |
| Andreas S._2 | 2022 | Metabolism and nutrition disorders | Hyperinsulinaemic hypoglycaemia | 1 | 304 | 302 | 0 |
| Andreas S._2 | 2022 | Metabolism and nutrition disorders | Hyperkalaemia | 1 | 304 | 302 | 0 |
| Andreas S._2 | 2022 | Metabolism and nutrition disorders | Hyponatraemia | 1 | 304 | 302 | 1 |
| Andreas S._2 | 2022 | Metabolism and nutrition disorders | Type 2 diabetes mellitus | 1 | 304 | 302 | 1 |
| Andreas S._2 | 2022 | Metabolism and nutrition disorders | Vitamin D deficiency | 0 | 304 | 302 | 1 |
| Andreas S._2 | 2022 | Musculoskeletal and connective tissue disorders | Arthralgia | 1 | 304 | 302 | 0 |
| Andreas S._2 | 2022 | Musculoskeletal and connective tissue disorders | Arthritis | 1 | 304 | 302 | 1 |
| Van Damme P._1 | 2019 | Musculoskeletal and connective tissue disorders | Back pain | 3 | 30 | 44 | 1 |
| Van Damme P._2 | 2019 | Musculoskeletal and connective tissue disorders | Back pain | 1 | 31 | 44 | 1 |
| Andreas S._2 | 2022 | Musculoskeletal and connective tissue disorders | Back pain | 1 | 304 | 302 | 1 |
| Andreas S._2 | 2022 | Musculoskeletal and connective tissue disorders | Cervical spinal stenosis | 0 | 304 | 302 | 1 |
| Andreas S._2 | 2022 | Musculoskeletal and connective tissue disorders | Costochondritis | 0 | 304 | 302 | 1 |
| Andreas S._2 | 2022 | Musculoskeletal and connective tissue disorders | Intervertebral disc degeneration | 1 | 304 | 302 | 0 |
| Andreas S._2 | 2022 | Musculoskeletal and connective tissue disorders | Intervertebral disc protrusion | 1 | 304 | 302 | 0 |
| Andreas S._2 | 2022 | Musculoskeletal and connective tissue disorders | Muscle spasms | 2 | 304 | 302 | 1 |
| Andreas S._2 | 2022 | Musculoskeletal and connective tissue disorders | Musculoskeletal chest pain | 0 | 304 | 302 | 1 |
| Andreas S._2 | 2022 | Musculoskeletal and connective tissue disorders | Musculoskeletal stiffness | 1 | 304 | 302 | 0 |
| Van Damme P._1 | 2019 | Musculoskeletal and connective tissue disorders | Myalgia | 12 | 30 | 44 | 6 |
| Van Damme P._2 | 2019 | Musculoskeletal and connective tissue disorders | Myalgia | 11 | 31 | 44 | 6 |
| Van Damme P._2 | 2019 | Musculoskeletal and connective tissue disorders | Myalgia | 9 | 31 | 44 | 3 |
| Van Damme P._1 | 2019 | Musculoskeletal and connective tissue disorders | Myalgia | 8 | 29 | 44 | 3 |
| Van Damme P._1 | 2019 | Musculoskeletal and connective tissue disorders | Myalgia | 7 | 30 | 44 | 4 |
| Van Damme P._2 | 2019 | Musculoskeletal and connective tissue disorders | Myalgia | 6 | 31 | 44 | 4 |
| Andreas S._1 | 2022 | Musculoskeletal and connective tissue disorders | Myalgia | 5 | 301 | 299 | 5 |
| Wilkinson T. M. A. | 2019 | Musculoskeletal and connective tissue disorders | Neck pain | 0 | 73 | 72 | 1 |
| Andreas S._2 | 2022 | Musculoskeletal and connective tissue disorders | Osteoarthritis | 2 | 304 | 302 | 2 |
| Andreas S._2 | 2022 | Musculoskeletal and connective tissue disorders | Pain in extremity | 1 | 304 | 302 | 4 |
| Andreas S._2 | 2022 | Musculoskeletal and connective tissue disorders | Rotator cuff syndrome | 1 | 304 | 302 | 0 |
| Andreas S._2 | 2022 | Musculoskeletal and connective tissue disorders | Spinal osteoarthritis | 0 | 304 | 302 | 1 |
| De Smedt P._1 | 2021 | Musculoskeletal and connective tissue disorders | Spinal stenosis | 1 | 26 | 28 | 0 |
| De Smedt P._2 | 2021 | Musculoskeletal and connective tissue disorders | Spinal stenosis | 0 | 27 | 28 | 0 |
| Andreas S._2 | 2022 | Musculoskeletal and connective tissue disorders | Synovial cyst | 1 | 304 | 302 | 0 |
| Andreas S._2 | 2022 | Musculoskeletal and connective tissue disorders | Tendon disorder | 0 | 304 | 302 | 1 |
| Andreas S._2 | 2022 | Musculoskeletal and connective tissue disorders | Tendonitis | 1 | 304 | 302 | 0 |
| Van Damme P._1 | 2019 | Musculoskeletal and connective tissue disorders | Tendonitis | 1 | 30 | 44 | 1 |
| Van Damme P._2 | 2019 | Musculoskeletal and connective tissue disorders | Tendonitis | 0 | 31 | 44 | 1 |
| Andreas S._2 | 2022 | Musculoskeletal and connective tissue disorders | Tenosynovitis | 0 | 304 | 302 | 1 |
| Andreas S._2 | 2022 | Musculoskeletal and connective tissue disorders | Tenosynovitis | 0 | 304 | 302 | 2 |
| Andreas S._2 | 2022 | Neoplasms benign, malignant and unspecified (incl cysts and polyps) | Adenocarcinoma gastric | 1 | 304 | 302 | 0 |
| Andreas S._2 | 2022 | Neoplasms benign, malignant and unspecified (incl cysts and polyps) | Adenocarcinoma of colon | 0 | 304 | 302 | 2 |
| Wilkinson T. M. A. | 2019 | Neoplasms benign, malignant and unspecified (incl cysts and polyps) | Adenocarcinoma | 0 | 73 | 72 | 1 |
| Andreas S._2 | 2022 | Neoplasms benign, malignant and unspecified (incl cysts and polyps) | Colon cancer | 1 | 304 | 302 | 0 |
| Andreas S._2 | 2022 | Neoplasms benign, malignant and unspecified (incl cysts and polyps) | Hepatic cancer | 0 | 304 | 302 | 1 |
| Andreas S._2 | 2022 | Neoplasms benign, malignant and unspecified (incl cysts and polyps) | Hypopharyngeal cancer stage IV | 1 | 304 | 302 | 0 |
| Andreas S._2 | 2022 | Neoplasms benign, malignant and unspecified (incl cysts and polyps) | Laryngeal cancer | 0 | 304 | 302 | 1 |
| Andreas S._2 | 2022 | Neoplasms benign, malignant and unspecified (incl cysts and polyps) | Leukaemia | 0 | 304 | 302 | 1 |
| De Smedt P._2 | 2021 | Neoplasms benign, malignant and unspecified (incl cysts and polyps) | Lung neoplasm malignant | 1 | 27 | 28 | 0 |
| De Smedt P._1 | 2021 | Neoplasms benign, malignant and unspecified (incl cysts and polyps) | Lung neoplasm malignant | 0 | 26 | 28 | 0 |
| Andreas S._2 | 2022 | Neoplasms benign, malignant and unspecified (incl cysts and polyps) | Lung neoplasm malignant | 0 | 304 | 302 | 2 |
| Wilkinson T. M. A. | 2019 | Neoplasms benign, malignant and unspecified (incl cysts and polyps) | Metastatic bronchial carcinoma | 0 | 73 | 72 | 1 |
| Andreas S._2 | 2022 | Neoplasms benign, malignant and unspecified (incl cysts and polyps) | Metastatic gastric cancer | 1 | 304 | 302 | 0 |
| Andreas S._2 | 2022 | Neoplasms benign, malignant and unspecified (incl cysts and polyps) | Ovarian cancer | 1 | 304 | 302 | 0 |
| Andreas S._2 | 2022 | Neoplasms benign, malignant and unspecified (incl cysts and polyps) | Pancreatic carcinoma | 1 | 304 | 302 | 0 |
| Andreas S._2 | 2022 | Neoplasms benign, malignant and unspecified (incl cysts and polyps) | Penile cancer | 1 | 304 | 302 | 0 |
| Andreas S._2 | 2022 | Neoplasms benign, malignant and unspecified (incl cysts and polyps) | Prostate cancer | 2 | 304 | 302 | 0 |
| De Smedt P._2 | 2021 | Neoplasms benign, malignant and unspecified (incl cysts and polyps) | Schwannoma | 1 | 27 | 28 | 0 |
| De Smedt P._1 | 2021 | Neoplasms benign, malignant and unspecified (incl cysts and polyps) | Schwannoma | 0 | 26 | 28 | 0 |
| Andreas S._2 | 2022 | Neoplasms benign, malignant and unspecified (incl cysts and polyps) | Squamous cell carcinoma of lung | 1 | 304 | 302 | 0 |
| Andreas S._2 | 2022 | Neoplasms benign, malignant and unspecified (incl cysts and polyps) | Squamous cell carcinoma of skin | 1 | 304 | 302 | 0 |
| Andreas S._2 | 2022 | Neoplasms benign, malignant and unspecified (incl cysts and polyps) | Squamous cell carcinoma of skin | 0 | 304 | 302 | 1 |
| Andreas S._2 | 2022 | Neoplasms benign, malignant and unspecified (incl cysts and polyps) | Transitional cell carcinoma | 0 | 304 | 302 | 1 |
| Wilkinson T. M. A. | 2019 | Neoplasms benign, malignant and unspecified (incl cysts and polyps) | Oesophageal squamous cell carcinoma | 1 | 73 | 72 | 0 |
| Andreas S._2 | 2022 | Nervous system disorders | Cerebrospinal fluid leakage | 0 | 304 | 302 | 1 |
| Andreas S._2 | 2022 | Nervous system disorders | Cerebrovascular accident | 1 | 304 | 302 | 0 |
| Andreas S._2 | 2022 | Nervous system disorders | Dizziness | 1 | 304 | 302 | 2 |
| Andreas S._2 | 2022 | Nervous system disorders | Epilepsy | 1 | 304 | 302 | 0 |
| Andreas S._2 | 2022 | Nervous system disorders | Focal dyscognitive seizures | 0 | 304 | 302 | 1 |
| Andreas S._2 | 2022 | Nervous system disorders | Guillain-Barre syndrome | 1 | 304 | 302 | 0 |
| Andreas S._2 | 2022 | Nervous system disorders | Haemorrhage intracranial | 1 | 304 | 302 | 1 |
| Andreas S._2 | 2022 | Nervous system disorders | Hemiplegic migraine | 1 | 304 | 302 | 0 |
| De Smedt P._1 | 2021 | Nervous system disorders | Ischaemic stroke | 1 | 26 | 28 | 0 |
| De Smedt P._2 | 2021 | Nervous system disorders | Ischaemic stroke | 0 | 27 | 28 | 0 |
| Andreas S._2 | 2022 | Nervous system disorders | Lumbar radiculopathy | 1 | 304 | 302 | 0 |
| Andreas S._2 | 2022 | Nervous system disorders | Paraesthesia | 0 | 304 | 302 | 1 |
| Andreas S._2 | 2022 | Nervous system disorders | Post herpetic neuralgia | 0 | 304 | 302 | 1 |
| Andreas S._2 | 2022 | Nervous system disorders | Post polio syndrome | 0 | 304 | 302 | 1 |
| Andreas S._2 | 2022 | Nervous system disorders | Ruptured cerebral aneurysm | 1 | 304 | 302 | 0 |
| Andreas S._2 | 2022 | Nervous system disorders | Subarachnoid haemorrhage | 0 | 304 | 302 | 1 |
| Andreas S._2 | 2022 | Nervous system disorders | Syncope | 2 | 304 | 302 | 0 |
| Andreas S._2 | 2022 | Nervous system disorders | Syncope | 1 | 304 | 302 | 0 |
| Andreas S._2 | 2022 | Nervous system disorders | Tension headache | 1 | 304 | 302 | 0 |
| Andreas S._2 | 2022 | Nervous system disorders | Transient ischaemic attack | 3 | 304 | 302 | 0 |
| Andreas S._2 | 2022 | Nervous system disorders | Tremor | 1 | 304 | 302 | 0 |
| Wilkinson T. M. A. | 2019 | Nervous system disorders | Dementia | 0 | 73 | 72 | 1 |
| Wilkinson T. M. A. | 2019 | Nervous system disorders | Facial paralysis | 1 | 73 | 72 | 0 |
| Andreas S._2 | 2022 | Nervous system disorders -Infections and infestations | CNS ventriculitis | 1 | 304 | 302 | 0 |
| Andreas S._2 | 2022 | Oral-infections and infestations | Gingivitis | 1 | 304 | 302 | 0 |
| Andreas S._2 | 2022 | Oral-infections and infestations | Oral candidiasis | 2 | 304 | 302 | 1 |
| Andreas S._2 | 2022 | Oral-infections and infestations | Oropharyngeal candidiasis | 1 | 304 | 302 | 0 |
| Andreas S._2 | 2022 | Product issues | Device loosening | 0 | 304 | 302 | 1 |
| Andreas S._2 | 2022 | Psychiatric disorders | Alcohol withdrawal syndrome | 0 | 304 | 302 | 2 |
| Andreas S._2 | 2022 | Psychiatric disorders | Anxiety | 2 | 304 | 302 | 2 |
| Andreas S._2 | 2022 | Psychiatric disorders | Anxiety | 0 | 304 | 302 | 1 |
| Andreas S._2 | 2022 | Psychiatric disorders | Depression | 2 | 304 | 302 | 0 |
| Andreas S._2 | 2022 | Psychiatric disorders | Drug withdrawal syndrome | 0 | 304 | 302 | 1 |
| Van Damme P._1 | 2019 | Psychiatric disorders | Insomnia | 0 | 30 | 44 | 1 |
| Van Damme P._2 | 2019 | Psychiatric disorders | Insomnia | 0 | 31 | 44 | 1 |
| Andreas S._2 | 2022 | Psychiatric disorders | Panic attack | 0 | 304 | 302 | 1 |
| Wilkinson T. M. A. | 2019 | Psychiatric disorders | Suicidal ideation | 1 | 73 | 72 | 0 |
| Andreas S._2 | 2022 | Renal and urinary disorders | Acute kidney injury | 1 | 304 | 302 | 1 |
| Andreas S._2 | 2022 | Renal and urinary disorders | Chromaturia | 0 | 304 | 302 | 1 |
| Andreas S._2 | 2022 | Renal and urinary disorders | Haematuria | 1 | 304 | 302 | 0 |
| Andreas S._2 | 2022 | Renal and urinary disorders | Nephrolithiasis | 1 | 304 | 302 | 0 |
| De Smedt P._1 | 2021 | Renal and urinary disorders | Nephrolithiasis | 0 | 26 | 28 | 1 |
| De Smedt P._2 | 2021 | Renal and urinary disorders | Nephrolithiasis | 0 | 27 | 28 | 1 |
| Van Damme P._1 | 2019 | Renal and urinary disorders | Sterile pyuria | 0 | 30 | 44 | 0 |
| Van Damme P._2 | 2019 | Renal and urinary disorders | Sterile pyuria | 0 | 31 | 44 | 0 |
| Andreas S._2 | 2022 | Renal and urinary disorders | Urinary incontinence | 1 | 304 | 302 | 0 |
| Andreas S._2 | 2022 | Renal and urinary disorders | Urine odour abnormal | 0 | 304 | 302 | 1 |
| Andreas S._2 | 2022 | Renal and urinary disorders-Infections and infestations | Cystitis | 3 | 304 | 302 | 1 |
| Andreas S._2 | 2022 | Renal and urinary disorders-Infections and infestations | Kidney infection | 0 | 304 | 302 | 1 |
| Andreas S._2 | 2022 | Renal and urinary disorders-Infections and infestations | Pyelonephritis | 0 | 304 | 302 | 1 |
| Wilkinson T. M. A. | 2019 | Renal and urinary disorders-Infections and infestations | Pyelonephritis | 1 | 73 | 72 | 0 |
| Andreas S._2 | 2022 | Renal and urinary disorders-Infections and infestations | Urinary tract infection | 1 | 304 | 302 | 4 |
| Wilkinson T. M. A. | 2019 | Renal and urinary disorders-Infections and infestations | Urinary tract infection | 1 | 73 | 72 | 4 |
| Andreas S._2 | 2022 | Renal and urinary disorders-Infections and infestations | Urinary tract infection | 0 | 304 | 302 | 3 |
| Wilkinson T. M. A. | 2019 | Renal and urinary disorders-Infections and infestations | Urinary tract infection | 1 | 73 | 72 | 4 |
| Andreas S._2 | 2022 | Renal and urinary disorders-Infections and infestations | Urosepsis | 0 | 304 | 302 | 2 |
| Andreas S._2 | 2022 | Reproductive system and breast disorders | Bartholin's cyst | 0 | 304 | 302 | 1 |
| Andreas S._2 | 2022 | Reproductive system and breast disorders | Breast mass | 0 | 304 | 302 | 1 |
| Andreas S._2 | 2022 | Reproductive system and breast disorders | Scrotal inflammation | 0 | 304 | 302 | 1 |
| Andreas S._2 | 2022 | Reproductive system and breast disorders | Scrotal pain | 1 | 304 | 302 | 0 |
| Andreas S._2 | 2022 | Reproductive system and breast disorders | Scrotal swelling | 1 | 304 | 302 | 0 |
| Andreas S._2 | 2022 | Respiratory, thoracic and mediastinal disorders | Acute pulmonary oedema | 0 | 304 | 302 | 1 |
| Wilkinson T. M. A. | 2019 | Respiratory, thoracic and mediastinal disorders | Acute pulmonary oedema | 0 | 73 | 72 | 1 |
| Andreas S._2 | 2022 | Respiratory, thoracic and mediastinal disorders | Acute respiratory distress syndrome | 1 | 304 | 302 | 0 |
| Andreas S._2 | 2022 | Respiratory, thoracic and mediastinal disorders | Acute respiratory failure | 7 | 304 | 302 | 7 |
| Andreas S._2 | 2022 | Respiratory, thoracic and mediastinal disorders | Atelectasis | 1 | 304 | 302 | 0 |
| Andreas S._2 | 2022 | Respiratory, thoracic and mediastinal disorders | Bronchospasm | 1 | 304 | 302 | 0 |
| Andreas S._2 | 2022 | Respiratory, thoracic and mediastinal disorders | Chest discomfort | 2 | 304 | 302 | 2 |
| Andreas S._2 | 2022 | Respiratory, thoracic and mediastinal disorders | Chest pain | 0 | 304 | 302 | 1 |
| Andreas S._2 | 2022 | Respiratory, thoracic and mediastinal disorders | Chronic obstructive pulmonary disease | 1 | 304 | 302 | 1 |
| Wilkinson T. M. A. | 2019 | Respiratory, thoracic and mediastinal disorders | Chronic obstructive pulmonary disease | 4 | 73 | 72 | 10 |
| Wilkinson T. M. A. | 2019 | Respiratory, thoracic and mediastinal disorders | Cough | 5 | 73 | 72 | 1 |
| Andreas S._2 | 2022 | Respiratory, thoracic and mediastinal disorders | Cough | 0 | 304 | 302 | 2 |
| Andreas S._2 | 2022 | Respiratory, thoracic and mediastinal disorders | Dyspnoea | 5 | 304 | 302 | 3 |
| Andreas S._2 | 2022 | Respiratory, thoracic and mediastinal disorders | Dyspnoea | 1 | 304 | 302 | 1 |
| Andreas S._2 | 2022 | Respiratory, thoracic and mediastinal disorders | Epistaxis | 2 | 304 | 302 | 0 |
| Andreas S._2 | 2022 | Respiratory, thoracic and mediastinal disorders | Epistaxis | 0 | 304 | 302 | 1 |
| Andreas S._2 | 2022 | Respiratory, thoracic and mediastinal disorders | Haemoptysis | 1 | 304 | 302 | 0 |
| Andreas S._2 | 2022 | Respiratory, thoracic and mediastinal disorders | Hypoxia | 1 | 304 | 302 | 1 |
| Andreas S._2 | 2022 | Respiratory, thoracic and mediastinal disorders | Increased upper airway secretion | 1 | 304 | 302 | 0 |
| Wilkinson T. M. A. | 2019 | Respiratory, thoracic and mediastinal disorders | Lung disorder | 0 | 73 | 72 | 1 |
| Andreas S._2 | 2022 | Respiratory, thoracic and mediastinal disorders | Nasal congestion | 3 | 304 | 302 | 0 |
| Van Damme P._1 | 2019 | Respiratory, thoracic and mediastinal disorders | Nasal obstruction | 0 | 30 | 44 | 0 |
| Van Damme P._2 | 2019 | Respiratory, thoracic and mediastinal disorders | Nasal obstruction | 0 | 31 | 44 | 0 |
| Andreas S._2 | 2022 | Respiratory, thoracic and mediastinal disorders | Nasal polyps | 0 | 304 | 302 | 1 |
| Van Damme P._1 | 2019 | Respiratory, thoracic and mediastinal disorders | Nasal septum deviation | 1 | 30 | 44 | 0 |
| Van Damme P._2 | 2019 | Respiratory, thoracic and mediastinal disorders | Nasal septum deviation | 0 | 31 | 44 | 0 |
| Andreas S._2 | 2022 | Respiratory, thoracic and mediastinal disorders | Non-cardiac chest pain | 1 | 304 | 302 | 0 |
| Andreas S._2 | 2022 | Respiratory, thoracic and mediastinal disorders | Non-cardiac chest pain | 0 | 304 | 302 | 1 |
| Wilkinson T. M. A. | 2019 | Respiratory, thoracic and mediastinal disorders | Non-cardiac chest pain | 1 | 73 | 72 | 0 |
| Andreas S._2 | 2022 | Respiratory, thoracic and mediastinal disorders | Paranasal sinus discomfort | 0 | 304 | 302 | 1 |
| Andreas S._2 | 2022 | Respiratory, thoracic and mediastinal disorders | Pharyngeal erythema | 1 | 304 | 302 | 0 |
| Andreas S._2 | 2022 | Respiratory, thoracic and mediastinal disorders | Pleuritic pain | 1 | 304 | 302 | 0 |
| Andreas S._2 | 2022 | Respiratory, thoracic and mediastinal disorders | Pneumothorax | 1 | 304 | 302 | 1 |
| Andreas S._2 | 2022 | Respiratory, thoracic and mediastinal disorders | Pulmonary arterial hypertension | 0 | 304 | 302 | 1 |
| Andreas S._2 | 2022 | Respiratory, thoracic and mediastinal disorders | Pulmonary embolism | 0 | 304 | 302 | 2 |
| Andreas S._2 | 2022 | Respiratory, thoracic and mediastinal disorders | Pulmonary fibrosis | 0 | 304 | 302 | 1 |
| Andreas S._2 | 2022 | Respiratory, thoracic and mediastinal disorders | Pulmonary mass | 1 | 304 | 302 | 0 |
| Andreas S._2 | 2022 | Respiratory, thoracic and mediastinal disorders | Pulmonary mass | 1 | 304 | 302 | 0 |
| Andreas S._2 | 2022 | Respiratory, thoracic and mediastinal disorders | Respiratory failure | 2 | 304 | 302 | 4 |
| Wilkinson T. M. A. | 2019 | Respiratory, thoracic and mediastinal disorders | Respiratory failure | 1 | 73 | 72 | 0 |
| Andreas S._2 | 2022 | Respiratory, thoracic and mediastinal disorders | Rhinitis allergic | 1 | 304 | 302 | 0 |
| Andreas S._2 | 2022 | Respiratory, thoracic and mediastinal disorders | Rhinorrhoea | 0 | 304 | 302 | 1 |
| Andreas S._2 | 2022 | Respiratory, thoracic and mediastinal disorders | Sinus congestion | 1 | 304 | 302 | 1 |
| Andreas S._2 | 2022 | Respiratory, thoracic and mediastinal disorders | Sleep apnoea syndrome | 1 | 304 | 302 | 0 |
| Andreas S._2 | 2022 | Respiratory, thoracic and mediastinal disorders | Sputum discoloured | 1 | 304 | 302 | 0 |
| Andreas S._2 | 2022 | Respiratory, thoracic and mediastinal disorders | Sputum increased | 0 | 304 | 302 | 1 |
| Wilkinson T. M. A. | 2019 | Respiratory, thoracic and mediastinal disorders | Systemic inflammatory response syndrome | 0 | 73 | 72 | 1 |
| Andreas S._2 | 2022 | Respiratory, thoracic and mediastinal disorders-Infections and infestations | Acute sinusitis | 1 | 304 | 302 | 1 |
| Andreas S._2 | 2022 | Respiratory, thoracic and mediastinal disorders-Infections and infestations | Bronchiectasis | 1 | 304 | 302 | 0 |
| Andreas S._2 | 2022 | Respiratory, thoracic and mediastinal disorders-Infections and infestations | Bronchitis | 1 | 304 | 302 | 1 |
| Andreas S._2 | 2022 | Respiratory, thoracic and mediastinal disorders-Infections and infestations | Bronchitis | 0 | 304 | 302 | 2 |
| Andreas S._2 | 2022 | Respiratory, thoracic and mediastinal disorders-Infections and infestations | Bronchitis chronic | 2 | 304 | 302 | 0 |
| Wilkinson T. M. A. | 2019 | Respiratory, thoracic and mediastinal disorders-Infections and infestations | Bronchitis viral | 1 | 73 | 72 | 0 |
| Andreas S._2 | 2022 | Respiratory, thoracic and mediastinal disorders-Infections and infestations | Chest injury | 1 | 304 | 302 | 1 |
| Andreas S._2 | 2022 | Respiratory, thoracic and mediastinal disorders-Infections and infestations | Chronic sinusitis | 0 | 304 | 302 | 1 |
| Andreas S._2 | 2022 | Respiratory, thoracic and mediastinal disorders-Infections and infestations | Lower respiratory tract infection | 1 | 304 | 302 | 0 |
| Wilkinson T. M. A. | 2019 | Respiratory, thoracic and mediastinal disorders-Infections and infestations | Lower respiratory tract infection | 1 | 73 | 72 | 1 |
| Andreas S._2 | 2022 | Respiratory, thoracic and mediastinal disorders-Infections and infestations | Metapneumovirus infection | 1 | 304 | 302 | 0 |
| Clancy R. | 1985 | Respiratory, thoracic and mediastinal disorders-Infections and infestations | Number of Patients with Lower respiratory infections | 1 | 17 | 16 | 9 |
| Clancy R. | 1985 | Respiratory, thoracic and mediastinal disorders-Infections and infestations | Number of Patients with Lower respiratory infections | 1 | 17 | 17 | 11 |
| Andreas S._2 | 2022 | Respiratory, thoracic and mediastinal disorders-Infections and infestations | Pneumonia pneumococcal | 2 | 304 | 302 | 0 |
| Andreas S._2 | 2022 | Respiratory, thoracic and mediastinal disorders-Infections and infestations | Pneumonia pseudomonal | 1 | 304 | 302 | 0 |
| Andreas S._2 | 2022 | Respiratory, thoracic and mediastinal disorders-Infections and infestations | Pneumonia staphylococcal | 0 | 304 | 302 | 1 |
| Andreas S._2 | 2022 | Respiratory, thoracic and mediastinal disorders-Infections and infestations | Respiratory syncytial virus infection | 0 | 304 | 302 | 1 |
| Andreas S._2 | 2022 | Respiratory, thoracic and mediastinal disorders-Infections and infestations | Respiratory tract infection | 1 | 304 | 302 | 1 |
| Andreas S._2 | 2022 | Respiratory, thoracic and mediastinal disorders-Infections and infestations | Sinusitis | 0 | 304 | 302 | 4 |
| Andreas S._2 | 2022 | Respiratory, thoracic and mediastinal disorders-Infections and infestations | Tracheobronchitis | 1 | 304 | 302 | 0 |
| Andreas S._2 | 2022 | Skin and subcutaneous tissue disorders | Dandruff | 1 | 304 | 302 | 0 |
| Andreas S._2 | 2022 | Skin and subcutaneous tissue disorders | Dermal cyst | 1 | 304 | 302 | 0 |
| Andreas S._2 | 2022 | Skin and subcutaneous tissue disorders | Dermatitis | 0 | 304 | 302 | 2 |
| Andreas S._2 | 2022 | Skin and subcutaneous tissue disorders | Eczema | 0 | 304 | 302 | 1 |
| Andreas S._2 | 2022 | Skin and subcutaneous tissue disorders | Erythema | 1 | 304 | 302 | 0 |
| Andreas S._2 | 2022 | Skin and subcutaneous tissue disorders | Photosensitivity reaction | 1 | 304 | 302 | 0 |
| Andreas S._2 | 2022 | Skin and subcutaneous tissue disorders | Pruritus | 0 | 304 | 302 | 1 |
| Andreas S._2 | 2022 | Skin and subcutaneous tissue disorders | Pruritus allergic | 1 | 304 | 302 | 0 |
| Andreas S._2 | 2022 | Skin and subcutaneous tissue disorders | Psoriasis | 1 | 304 | 302 | 0 |
| Andreas S._2 | 2022 | Skin and subcutaneous tissue disorders | Skin lesion | 1 | 304 | 302 | 0 |
| Andreas S._2 | 2022 | Vascular disorders | Aortic aneurysm | 1 | 304 | 302 | 1 |
| Andreas S._2 | 2022 | Vascular disorders | Femoral artery aneurysm | 1 | 304 | 302 | 0 |
| Wilkinson T. M. A. | 2019 | Vascular disorders | Haematoma | 1 | 73 | 72 | 0 |
| Andreas S._2 | 2022 | Vascular disorders | Hypertension | 1 | 304 | 302 | 1 |
| Andreas S._2 | 2022 | Vascular disorders | Hypotension | 0 | 304 | 302 | 1 |
| Andreas S._2 | 2022 | Vascular disorders | Peripheral arterial occlusive disease | 0 | 304 | 302 | 1 |
| Andreas S._2 | 2022 | Vascular disorders | Peripheral artery aneurysm | 1 | 304 | 302 | 0 |
| Andreas S._2 | 2022 | Vascular disorders | Peripheral vein occlusion | 0 | 304 | 302 | 1 |
| Andreas S._2 | 2022 | Vascular disorders | Peripheral venous disease | 1 | 304 | 302 | 0 |
| Andreas S._2 | 2022 | Vascular disorders | Thrombophlebitis | 0 | 304 | 302 | 1 |

N, sample size; No., number of.

## Figure S1. Assessment of risk of bias.


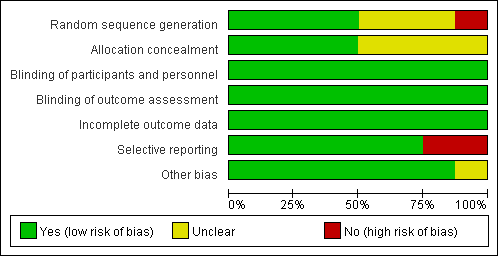


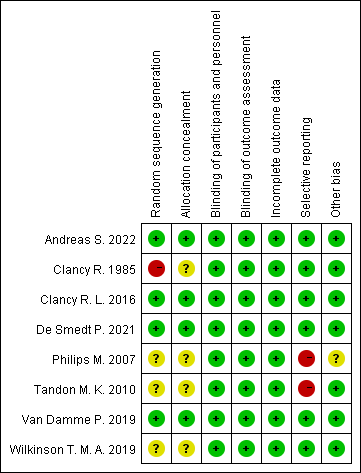


## Figure S2. The sensitivity analysis of AECOPD.


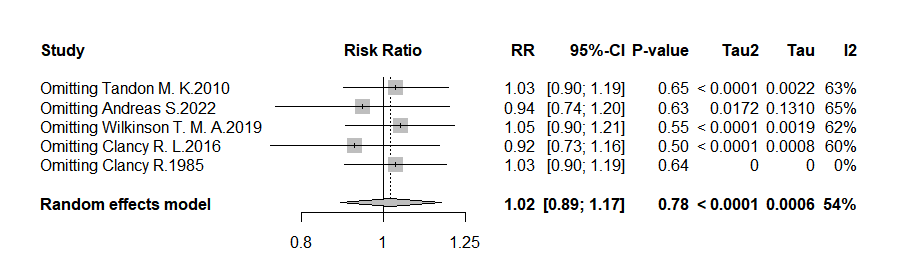


## Figure S3. The sensitivity analysis of all-cause mortality.


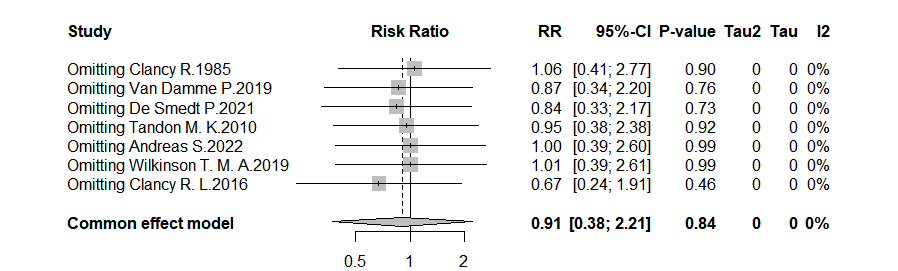


## Figure S4. The sensitivity analysis of hospitalization.


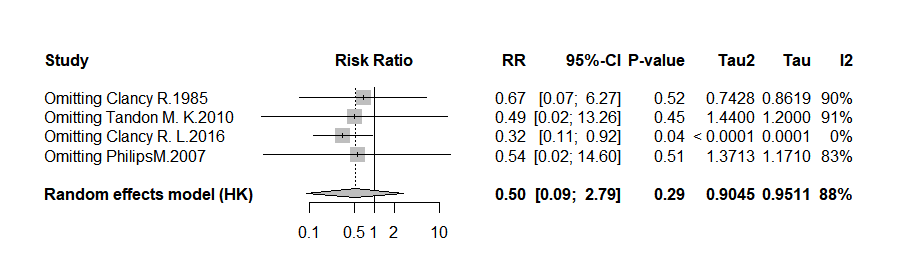


## Figure S5. The sensitivity analysis of serious adverse events.

**
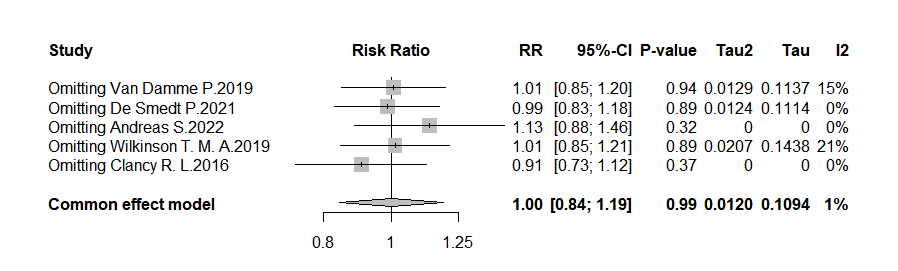
**

## Figure S6. The sensitivity analysis of grade 3 serious adverse events.


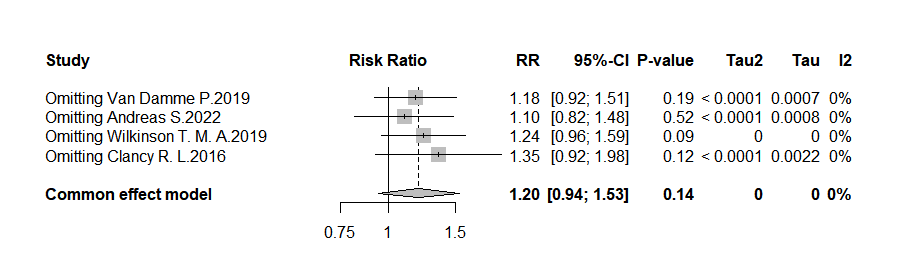


## Figure S7. The sensitivity analysis of pIMDs.


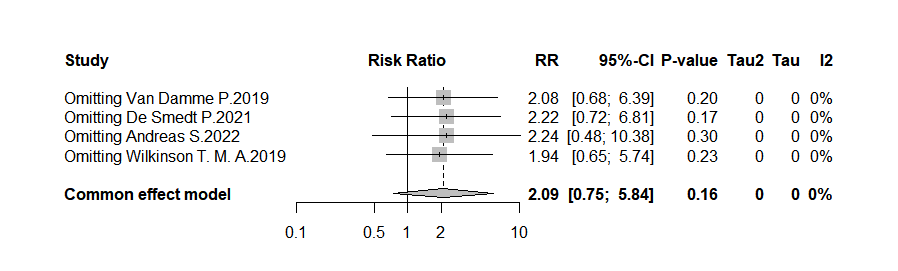

Supplement: Supplementary file 1 [file Supplementary_file_1.docx]
